# Patterns, biases and prospects in the distribution and diversity of Neotropical snakes

**DOI:** 10.1111/geb.12679

**Published:** 2017-11-23

**Authors:** Thaís B. Guedes, Ricardo J. Sawaya, Alexander Zizka, Shawn Laffan, Søren Faurby, R. Alexander Pyron, Renato S. Bérnils, Martin Jansen, Paulo Passos, Ana L. C. Prudente, Diego F. Cisneros‐Heredia, Henrique B. Braz, Cristiano de C. Nogueira, Alexandre Antonelli, Shai Meiri

**Affiliations:** ^1^ Department of Biological and Environmental Sciences University of Gothenburg Göteborg Sweden; ^2^ Gothenburg Global Biodiversity Center Göteborg Sweden; ^3^ Departamento de Ecologia e Biologia Evolutiva Universidade Federal de São Paulo (UNIFESP) Diadema São Paulo Brazil; ^4^ Laboratório de Herpetologia Museu de Zoologia da Universidade de São Paulo (MZUSP) São Paulo São Paulo Brazil; ^5^ Centre for Ecosystem Science, School of Biological, Earth and Environmental Sciences The University of New South Wales Sydney New South Wales Australia; ^6^ Department of Biological Sciences The George Washington University Washington District of Columbia, U.S.A.; ^7^ Universidade Federal do Espírito Santo (UFES), Campus Litorâneo São Mateus Espírito Santo Brazil; ^8^ Section of Herpetology Senckenberg Research Institute and Nature Museum Frankfurt am Main Germany; ^9^ Departamento de Vertebrados, Museu Nacional (MNRJ) Universidade Federal do Rio de Janeiro Rio de Janeiro Rio de Janeiro Brazil; ^10^ Departamento de Zoologia Laboratório de Herpetologia, Museu Paraense Emilio Goeldi (MPEG) Belém Pará Brazil; ^11^ Laboratorio de Zoología Terrestre, Colegio de Ciencias Biológicas y Ambientales (COCIBA) Universidad San Francisco de Quito (USFQ) Quito Ecuador; ^12^ Department of Geography King's College London Strand London United Kingdom; ^13^ The Natural History Museum (UK), Kensington London United Kingdom; ^14^ School of Life and Environmental Sciences, Faculty of Science The University of Sydney Sydney New South Wales Australia; ^15^ Departamento de Ecologia Universidade de São Paulo (USP) São Paulo São Paulo Brazil; ^16^ Gothenburg Botanical Garden Göteborg Sweden

**Keywords:** conservation, data availability, GBIF, geographical distribution, phylogenetic diversity, sampling gaps, Serpentes, species richness

## Abstract

**Motivation:**

We generated a novel database of Neotropical snakes (one of the world's richest herpetofauna) combining the most comprehensive, manually compiled distribution dataset with publicly available data. We assess, for the first time, the diversity patterns for all Neotropical snakes as well as sampling density and sampling biases.

**Main types of variables contained:**

We compiled three databases of species occurrences: a dataset downloaded from the Global Biodiversity Information Facility (GBIF), a verified dataset built through taxonomic work and specialized literature, and a combined dataset comprising a cleaned version of the GBIF dataset merged with the verified dataset.

**Spatial location and grain:**

Neotropics, Behrmann projection equivalent to 1° × 1°.

**Time period:**

Specimens housed in museums during the last 150 years.

**Major taxa studied:**

Squamata: Serpentes.

**Software format:**

Geographical information system (GIS).

**Results:**

The combined dataset provides the most comprehensive distribution database for Neotropical snakes to date. It contains 147,515 records for 886 species across 12 families, representing 74% of all species of snakes, spanning 27 countries in the Americas. Species richness and phylogenetic diversity show overall similar patterns. Amazonia is the least sampled Neotropical region, whereas most well‐sampled sites are located near large universities and scientific collections. We provide a list and updated maps of geographical distribution of all snake species surveyed.

**Main conclusions:**

The biodiversity metrics of Neotropical snakes reflect patterns previously documented for other vertebrates, suggesting that similar factors may determine the diversity of both ectothermic and endothermic animals. We suggest conservation strategies for high‐diversity areas and sampling efforts be directed towards Amazonia and poorly known species.

## 1 INTRODUCTION

Reptiles are a highly diverse group of terrestrial vertebrates with 10,450 known species, with this number increasing at *c*. 100 per year (Tonini, Beard, Ferreira, Jetz, & Pyron, 2016; Uetz & Hošek, 2016). It is probably the most neglected group in conservation prioritizations, as only 52% of the described species have been assessed in the International Union for Conservation of Nature (IUCN) Red List of Threatened Species (IUCN, 2017). Most of the assessed species have been categorized based on range size, of which 20% (Böhm, Collen, & Baillie, 2013) are considered data deficient owing to the lack of appropriate data on taxonomy, ecology, distribution, population trends and threats (Bland & Böhm, 2016; Böhm et al., 2013). This contrasts with that for other vertebrates, as for instance only 0.6% of birds and 15% of mammals are data deficient (Butchart & Bird, 2010; Schipper et al., 2008).

Among reptiles, there are *c*. 3,500 snake species globally, inhabiting temperate to tropical environments, in terrestrial and aquatic habitats (Uetz & Hošek, 2016; Wallach, Williams, & Boundy, 2014). As for most reptiles, distribution data for snake species remain scarce, and consequently, they are excluded from most large‐scale studies of biodiversity and distribution patterns (e.g., Jenkins, Alves, Uezu, & Vale, 2015; Moura, Villalobos, Costa, & Garcia, 2016). Although reliable estimates of snake diversity would contribute to global and regional strategies for biological conservation, no detailed data have yet been compiled for the Neotropics, despite it comprising one of the world's richest herpetofaunas (Böhm et al., 2013; Meiri & Chapple, 2016).

Here, we present a new database of snake occurrences covering the entire Neotropics and assess, for the first time, the diversity patterns for all Neotropical snakes as well as sampling artefacts. We hypothesize that snake diversity follows a similar pattern to those already described for other vertebrates in the Neotropics (Jenkins et al., 2015; Moura et al., 2016). We generated our novel database by combining the most comprehensive, manually compiled distribution dataset with publicly available data, from which we calculate species richness (SR) and phylogenetic diversity (PD) as well as sampling density and sampling biases. Finally, we discuss prospects for more informed conservation strategies and design research agendas.

## 2 MATERIAL AND METHODS

### 2.1 Data sources

We compiled three datasets for snakes recorded in the Neotropical region (sensu Olson et al., 2001), from central Mexico to southern South America, including all Caribbean islands. We included only records identified at the species level.

The raw dataset (RD) comprised georeferenced records for snakes downloaded from the Global Biodiversity Information Facility (GBIF; http://doi.org/10.15468/dl.tdwbqp). We filtered our search for records linked to specimens, literature occurrences and material samples, leaving out records lacking associated vouchers.

The verified dataset (VD) comprised geographical occurrences from vouchered specimens examined in natural history museums (Supporting Information Appendix S1) and required a large collaborative effort among herpetologists. The initial focus of the VD was to gather data on Brazilian snakes but also including their distribution outside the country. This was then expanded also to include species and records from other Latin American countries outside Brazil, through point occurrence data from vouchers and scientific literature.

The combined dataset (CD) was constructed by merging a cleaned version of the RD with the VD. To produce the RD cleaned dataset, we taxonomically validated and updated the species names. Geographical coordinates were cleaned by verification of geographical data and map compilation using the speciesgeocodeR (Töpel et al., 2017) and maptools (Lewin‐Koh et al., 2011) packages in R (R Core Team, 2017). A commented list containing all taxonomic and geographical changes applied to the RD in this process (without voucher verification) is provided in Supporting Information Appendix S2. Then, we merged the GBIF cleaned dataset with the VD to form the CD. We also removed from CD all redundant coordinates for each species (i.e., records with identical latitude and longitude values).

### 2.2 Analyses

#### 2.2.1 Species richness and phylogenetic diversity

**Figure 1:**
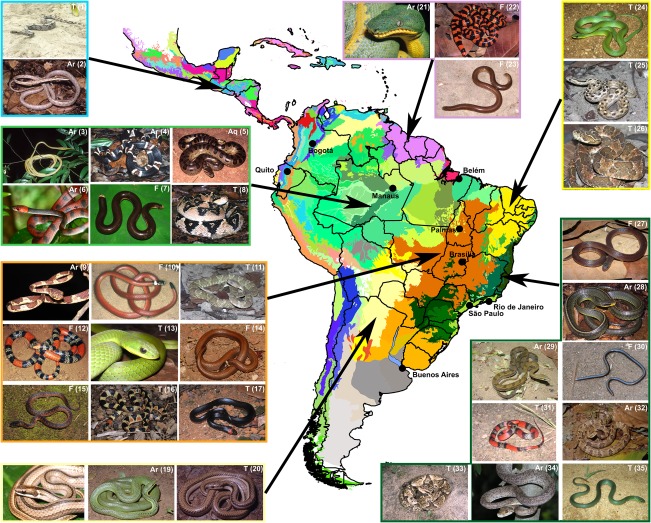
Neotropical region and ecoregion limits adopted here (sensu Olson et al., 2001), together with representative snakes species recorded for Central America Montane Forests: 1.1 *Boa constrictor*, 1.2 *Oxybelis aeneus*; Amazonia Most Forests: 1.3 *Philodryas argentea*, 1.4 *Rhinobothryum lentiginosum*, 1.5 *Eunectes murinus*, 1.6 *Siphlophis compressus*, 1.7 *Amerotyphlops reticulatus*, 1.8 *Lachesis muta*; Cerrado: 1.9 *Imantodes cenchoa*, 1.10 *Apostolepis flavotorquata*, 1.11 *Bothrops lutzi*, 1.12 *Micrurus frontalis*, 1.13 *Erythrolamprus typhlus*, 1.14 *Phalotris lativittatus*, 1.15 *Xenopholis undulatus*, 1.16 *Oxyrhopus rhombifer*, 1.17 *Rhachidelus brazili*; Chaco: 1.18 *Psomophis genimaculatus*, 1.19 *Philodryas baroni*, 1.20 *Phimophis vittatus*; Guianian Moist Forests: 1.21 *Corallus caninus*, 1.22 *Anilius scytale*, 1.23 *Amerotyphlops brongersmianus*; Caatinga: 1.24 *Erythrolamprus viridis*, 1.25 *Thamnodynastes phoenix*, 1.26 *Bothrops erythromelas*; and in the Atlantic Forest: 1.27 *Atractus maculatus*, 1.28 *Chironius bicarinatus*, 1.29 *Tropidodryas striaticeps*, 1.30 *Liotyphlops beui*, 1.31 *Oxyrhopus guibei*, 1.32 *Dipsas albifrons*, 1.33 *Bothrops jararaca*, 1.34 *Corallus hortulanus*, 1.35 *Erythrolamprus atraventer*. The abbreviations indicate common life habits of the Neotropical snakes: aquatic (Aq), arboreal (Ar), fossorial (F), terrestrial (T). Photograph credits: Cristiano C. Nogueira (10, 12), Crizanto C. Brito (27), Henrique B. Braz (14), Ivan Sazima (24, 35), Luiz C. Turci (7), Marcio Martins (4), Marco Sena (6), Martin Jansen (9, 13, 18, 23, 31), Otavio A. V. Marques (2, 3, 5, 15, 16, 17, 19, 20, 21, 22, 28, 30, 32), Ricardo J. Sawaya (33), Thaís B. Guedes (1, 8, 11, 25, 26, 29, 34)

We used the CD for species richness (SR) and phylogenetic diversity (PD) analyses. Both analyses were performed at two spatial resolutions: the grid cell scale, which was on an equal area Behrmann projection with 360 columns (corresponding to 1° × 1° at 30° N or 30° S, 1° × *c*. 0.75° at the equator and 1° × *c*. 9.5° at the poles), and the ecoregion scale corresponding to polygons (sensu Olson et al., 2001; Figure 1). We ran all analyses using the software Biodiverse, version 1.1 (Laffan, Lubarsky, & Rosauer, 2010).

Phylogenetic diversity and species richness are usually correlated (Morlon et al., 2011). However, SR takes into account only distribution data for each species, whereas PD is calculated by using distribution data plus branch lengths of the phylogeny. Thus, PD incorporates evolutionary history that is not expressed by SR (Faith, 1992, 2008; Tucker et al., 2017).

The PD analysis was based on distribution data and a sample of 100 trees, from which we calculated mean values for each grid cell and ecoregion polygons to synthesize the result of PD in a single map for each scale adopted. We used the phylogeny provided by Tonini et al. (2016). The variance in PD metrics across the sample of trees reported in their study was low; thus, we considered this a sufficient approximation of PD. Phylogenetic diversity analyses require a precise match between distribution data and the terminals of the phylogeny. Of the 886 species in the CD, 847 (96%) were present on the tree and were used for both SR and PD analyses to allow a more direct comparison between the two analyses.

#### 2.2.2 Sampling gaps

We calculated the number of occurrences across grid cells superimposed onto the Neotropical region to identify the intensity of sampling in each dataset.

## 3 RESULTS

### 3.1 Data availability

**Figure 2:**
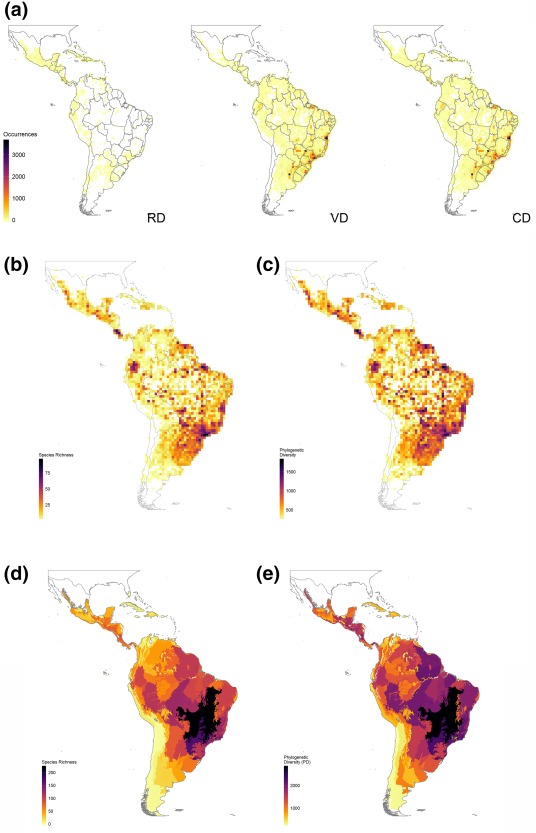
Species occurrence data and spatial patterns of Neotropical snake diversity. (a) Geographical coverage of sampling of snakes measured in 1° × 1° grid cells. RD = raw dataset, obtained from www.gbif.org; VD = verified dataset, presented here; CD = combined dataset, produced by merging RD and VD. (b) Species richness at grid cells. (c) Phylogenetic diversity at grid cells. (d) Species richness at the ecoregion scale. (e) Phylogenetic diversity at the ecoregion scale

The RD includes 7,299 records of 659 species of snakes from 12 families (Table 1). The records are distributed over 25 countries (Figure 2a). A large number of records were derived from Central America and the West Indies, whereas the data are especially poor and sparce in South America (Figure 2a).

**Table 1:** Number of species and amount of occurrence data in the three datasets of snakes recorded in the Neotropical region

Dataset	Number of occurrences	Number of species
Raw dataset	7,299	659
Verified dataset	140,368	488
Combined dataset	147,515	886

The VD contains almost 20 times more records than the RD. It includes 140,368 georeferenced records for 488 species from 10 families (Table 1). The records are distributed over 18 countries (Figure 2a), especially in South America, with 436 species recorded only in this region (Figure 2a).

We excluded 152 inconsistences in taxonomic and geographical cleaning of RD (Supporting Information Appendix S2). Thus, the CD has a total of 147,515 georeferenced records, representing 886 species in 12 families across 27 countries (Table 1 and Figure 2a). To our knowledge, this constitutes the most extensive and complete dataset of snake distributions for the Neotropical region, both in number of occurrences and number of species (Table 1 and Figure 2a). Maps for each species and a list of all Neotropical snake species included in this study, with their status of their known geographical distribution, are provided in Supporting Information Appendices S3 and S4.

### 3.2 Spatial patterns of species richness and phylogenetic diversity

#### 3.2.1 Grid cell diversity

SR and PD are spatially very similar (Figure 2b,c). Species richness (60–120 species) and PD (1,000–2,000) are highest in the Atlantic Forest of southeast Brazil, closely followed by the Amazonian region along some large cities or close to important rivers, the coastal forests in northernmost South America, the Andean forests of Ecuador, the moist and montane forests of Central America, the Cerrado savannas in Central Brazilian Plateau and nearby Tocantins drainage, and the Pantanal wetlands. Intermediate values of SR (30‐60 species) and PD (500‐1,000) are found in the semi‐arid Caatinga in northeast Brazil, in a large continuous (SR) or scattered (PD) area in the Cerrado savannas, the Pampas and Chaco regions in southern South America, in the Andean region over Colombia, Ecuador and Peru, and over a large portion in Central America.

For SR, values < 30 species are found in small portion of the Neotropical region (e.g., West Indies, north‐western Amazonia, northern and southern Bolivia, and north of the Chaco). For PD, a large number of cells show low values (between zero and 500), with the largest patches in Amazonia.

#### 3.2.2 Ecoregion diversity

We recorded species in 187 ecoregions (Figure 2d,e; Supporting Information Appendix S5). The Cerrado is the richest ecoregion, with occurrences of 222 species of snakes, and also has the highest PD value (2,700). However, the ecoregions inside the Atlantic Forest domain also presented high values of SR and PD. Ecoregions in the Caatinga, the extra‐Andean region from Colombia to Peru, Costa Rica, Chaco and Pampas have intermediate values of both SR and PD (Figure 2d,e; Supporting Information Appendix S5).

The ecoregions with lowest SR and PD are located in the southern part of the Andes and the West Indies islands. Thirty‐one ecoregions include records for just one to three species each (Figure 2d,e; Supporting Information Appendix S5).

### 3.3 Sampling gaps

Based on CD, the most poorly sampled Neotropical region is the Amazon, where all grid cells harbour < 500 records and 1,600,000 km^2^ have no records at all (Figure 2a). The Andean region is also poorly sampled, with 900,000 km^2^ empty and all others having < 500 records. The Lesser Antilles and Central America are also poorly sampled. The best‐sampled region is the Atlantic Forest (400,000 km^2^, containing 1,000–3,000 occurrences; Figure 2a). Some cells are well sampled, even though surrounding cells have very few records.

## 4 DISCUSSION

### 4.1 Data availability

We found errors associated with non‐updated nomenclature and erroneous georeferences in the RD. This reinforces previous suggestions (e.g., Ficetola et al., 2013; Maldonado et al., 2015; Meyer, Weigelt, & Kreft, 2016) that GBIF data should not be used without proper verification and cleaning. The verified dataset, albeit smaller in the absolute number of species and records outside Brazil, can be considered well curated. As these two datasets are so different in geographical and taxonomic representation, merging them proved to be a suitable approach. Combining the RD cleaned dataset with the verified dataset almost doubled the number of species in the CD and substantially increased the geographical coverage to cover the Neotropical region more adequately.

This study provides the most comprehensive and novel database on snakes in the Neotropical region to date. Our CD increased the knowledge about Neotropical snakes, providing data for 886 species, an improvement of 670 species compared with previous studies (216 species by Böhm et al., 2013). We believe that CD also led to a considerable reduction in the number of ‘poorly known’ snake species regarding geographical distribution, especially for the tropical regions known to contain the most species classified as data deficient or threatened (see Supporting Information Appendix S4; Bland & Böhm, 2016; Böhm et al., 2013; Tingley, Meiri, & Chapple, 2016).

### 4.2 Spatial patterns of species richness and phylogenetic diversity

The SR patterns found for Neotropical snakes broadly correspond to the patterns previously reported for other vertebrates (e.g., Fenker, Tedeschi, Pyron, & Nogueira, 2014; Jenkins et al., 2015; Moura et al., 2016). In contrast, our results contradict previous suggestions that Amazonia (here considered as poorly sampled) is the richest area for Neotropical reptiles (Böhm et al., 2013). This discrepancy may be explained by differences between our dataset and that of Böhm et al. (2013), which also included lizards, used species ranges instead of grid cells, adopted a different spatial scale, and was based on a random sampling, which in theory is meant to provide an adequate representation of species globally, but in practice may be problematical.

A different view of areas harbouring high SR and PD emerges on the scale of ecoregions (Figure 2d,e). For both indices, the Cerrado is the most diverse region. Accordingly, these results indicate that snake diversity in seasonally dry tropical forests may be more diverse than in rain forests, a pattern not previously inferred. The Cerrado is a global biodiversity hotspot (Mittermeier, Turner, Larsen, Brooks, & Gascon, 2011; Myers et al., 2000), harbouring ≥ 153 species of snakes, of which 49 are endemic (Guedes, Nogueira, & Marques, 2014). It is also the world's most species rich savanna in number of woody plant species and has higher diversity than any other dry forests in the Neotropics (DRYFLOR et al., 2016). However, our results could be biased by the ecoregion boundaries used here, which separated the Atlantic Forest into distinct subregions, but did not do so to the Cerrado. As a whole, the Atlantic Forest harbours the richest snake fauna, including 236 species, of which 83 are endemic (Guedes et al., 2014). This situation reinforces the importance of refined data on species distributions for assessing the influence of spatial scale on patterns of biodiversity.

Despite the close relationship between SR and PD, the most species‐rich areas are not fully coincident with areas of highest PD, as already reported by Fenker et al. (2014) for a clade of snakes. At the grid cell scale, we find highest SR and PD in forested areas, a similar pattern previously reported for amphibians, mammals and birds (Jenkins et al., 2015; Moura et al., 2016). Such areas also appear to contain high PD for particular groups of snakes, such as the relatively diverse genus, *Bothrops* (Fenker et al., 2014). However, high‐PD areas can probably be explained by sympatry of widely divergent lineages. This should occur in grid cells where species of open‐habitat clades are found together with forest‐adapted clades (Fenker et al., 2014).

### 4.3 Sampling gaps

Our sampling gap map reflects a situation similar to that documented for other vertebrates (Meyer, Kreft, Guralnick, & Jetz, 2015). Amazonia has the smallest number of records of snakes in relationship to its area, which was predictable in face of the scattered data already reported for other groups (Peres, 2005). The region's high inaccessibility, low investments in local research and the relative shortage of experts to explore this huge area are likely to explain this result. In contrast, well‐sampled areas were coincident with the location of the most active universities and scientific collections of reptiles.

## 5 CONCLUSIONS

Our study demonstrates that Neotropical snake diversity is unevenly distributed, with some ecoregions, such as the Cerrado, containing a disproportionately high diversity. We also showed that merging public and manually compiled data sources is likely to provide the largest taxonomic and geographical coverage for any system under study. However, a proper taxonomic verification, examination and assessment of biases of the public dataset proved crucial. As a result, we can now provide a solid and reliable foundation for any kind of meta‐analysis, including the assessment of climate change effects, conservation strategies or design of future research agendas. Conservation priorities should focus on areas of high diversity values as well as high threat by landscape changes. Finally, we found highest diversity values in forested areas, reinforcing the need for general habitat protection compared with actions that are targeting specific species.

In order to increase our knowledge about Neotropical snakes, a geographically and taxonomically focused sampling is required, targeting Amazonia and those species whose distributions are so far largely unknown.

## DATA ACCESSIBILITY

Maps containing the distribution of all species used in this study are available as rasterfile in Supporting Information Appendix S3.

## AUTHOR CONTRIBUTIONS

T.B.G. and A.A. conceived the research; T.B.G., R.J.S., R.S.B., M.J., P.P., A.L.C.P., D.F.C.‐H., H.B.B. and C.C.N. collected the data by visiting natural history museums and consulting literature; A.P. provided the phylogenies; T.B.G. analysed the biogeographical data and drew the maps, assisted by A.Z., S.L. and S.F.; T.B.G. and A.A. led the writing, with contributions from all authors, interpretation and discussion of the results.

BIOSKETCH **Thaís Barreto Guedes** (http://www.tbguedes.com) is an evolutionary biologist interested in biogeography, macroecology and conservation of the herpetofauna. She is investigating these subjects on a broad scale especially in snakes, but also in amphibians and other reptiles.

## Supporting information

Additional Supporting Information may be found online in the supporting information tab for this article.

Supporting Appendix S1

Supporting Appendix S2

Supporting Appendix S3

Supporting Appendix S4

Supporting Appendix S5
