# Supplementary material for: Patterns, biases and prospects in the distribution and diversity of Neotropical snakes
Source: Glob Ecol Biogeogr. 2017 Nov 23;27(1):14–21. doi: 10.1111/geb.12679 (PMC5765514; doi:10.1111/geb.12679)
Supplement: Supplementary file 1 — Supporting Appendix S1 [file GEB-27-14-s001.pdf]

Patterns, biases and prospects in the distribution and diversity of Neotropical snakes

Thaís B. Guedes, Ricardo J. Sawaya, Alexander Zizka, Shawn Laffan, Alexander Pyron, Renato S. Bérnils, Martin Jansen, Paulo Passos, Ana L. C. Prudente, Diego F. Cisneros-Heredia, Henrique B. Braz, Cristiano de C. Nogueira & Alexandre Antonelli

Appendix S1 List of natural history museums from where the specimens of snakes were examined and point-occurrence was collected to build the Verified Dataset (VD).

Acronym	Institution	City	Country
AMNH	American Museum of Natural History	New York	U.S.A.
ANSP	The Academy of Natural Sciences	Philadelphia	U.S.A.
BMNH	British Museum of Natural History	London	U.K.
CAS	California Academy of Science	San Franciso	U.S.A.
CBF	Colección Boliviana de Fauna	La Paz	Bolivia
CBMI	Colección de Referencia Itaipú Binacional	Ciudad del Este	Paraguay
CCG	Colección Carlos Grisolía	Buenos Aires	Argentina
CCNG	Coleção Carlos Nicolau Gofferjé, Universidade do Vale do Itajaí	Itajaí	Brazil
CENAI	Centro Nacional de Investigaciones Iológica	Buenos Aires	Argentina
CENAI/CHINM	Colección Herpetológica Instituto Nacional de Microbiología Carlos Malbrán	Buenos Aires	Argentina
CEPB	Coleção Herpetológica da Pontifícia Universidade Católica de Goiais	Goiania	Brazil
CEUCH	Centro de Estudos Universitários da Universidade Federal do Mato Grosso do Sul	Corumbá	Brazil
CFA	Colección "Félix de Azára" de Programa de Biología Basica	Corrientes	Argentina
CH-UNLS	Colección Herpetológica de la Universidad Nacional de San Luis	San Luis	Argentina
CHBEZ	Coleção Herpetológica da Universidade Federal do Rio Grande do Norte	Natal	Brazil
CHC	Colección Herpetológica Corrientes	Corrientes	Argentina

Acronym	Institution	City	Country
CHUFC	Museu na Universidade Federal do Ceará	Fortaleza	Brazil
CHUFS	Museu na Universidade Estadual de Sergipe	Aracajú	Brazil
CHUFSC	Universidade Federal de Santa Catarina	Florianópolis	Brazil
CHUNAM	Colección Herpetológica de la Universidad Nacional de Misiones	Posadas	Argentina
CHUNB	Coleção Herpetológica da Universidade de Brasília	Brasília	Brazil
CIES	Centro de Investigaciones Ecológicas Subtropicales	Puerto Iguazú	Argentina
CMNH	Carnegie Museum	Pitssburgh	U.S.A.
CRUPF	Museu Zoológico Augusto Ruschi, Universidade de Passo Fundo	Passo Fundo	Brazil
CVR	Colección de Vertebrados-Reptiles, Faculdad de Humanidades y Ciencias	Montevideo	Uruguay
CZA	Centro de Zoologia Aplicada	Córdoba	Argentina
CZGB	Coleção Zoologica Gregogrio Bondar, Universidade Estadual de Santa Cruz	Ilhéus	Brazil
CZNC	Coleção Zoológica Norte Capixaba	São Mateus	Brazil
DB	Diego Baldo Personal Collection housed at Museo de La Plata (MLP)	La Plata	Argentina
DFCH-USFQ0H	Diego Cisneros-Heredia Personal Collection housed at Universidad San Francisco de Quito	Quito	Ecuador
DHMECN	División de Herpetología, Museo Ecuatoriano de Ciencias Naturales	Quito	Ecuador
DZUFRJ	Departamento de Zoologia da Universidade Federal do Rio de Janeiro	Rio de Janeiro	Brazil
DZURGS	Departamento de Zoologia da Universidade Federal do Rio Grande do Sul	Porto Alegre	Brazil
EBD	Estación Ecológica Doñana	Seville	Spain
EBRG	Estación Biológica Rancho Grande	Maracaybo	Venezuela
EPN	Escuela Politécnica Nacional	Quito	Ecuador
FFCLRP	Faculdade de Filosofia, Ciências e Letras de Ribeirão Preto, Universidade de São Paulo	Ribeirão Preto	Brazil
FHGO	Fundación Herpetologica Gustavo Orces	Quito	Ecuador
FML	Fundación Miguel Lillo	San Miguel de Tucumán	Argentina
FMNH	Field Museum of Natural History	Chicago	U.S.A.
FMT	Fundação Medicina Tropical	Manaus	Brazil
FPR	Fundacion Puerto Rastrojo	Bogota	Colombia

Acronym	Institution	City	Country
FUNED	Coleção Herpetológica da Fundação Ezequiel Dias	Belo Horizonte	Brazil
FURB	Universidade Regional de Blumenau	Blumenau	Brazil
FURG	Universidade Federal do Rio Grande do Sul	Porto Alegre	Brazil
GOV	Gustavo Orcés-Villagomes Collection (Escuela Politécnica Nacional)	Quito	Ecuador
HCPAP	Coleçaõ de Vertebrados da Embrapa Pantanal	Corumbá	Brazil
HVB	Hospital Vital Brazil housed at Instituto Butantan	São Paulo	Brazil
IAvH	Instituto Alexander von Humbolt	Villa de Leyva	Colombia
IBSP	Instituto Butantan	São Paulo	Brazil
ICN	Instituto de Ciencias Naturales, Universidad Nacional de Colombia	Bogotá	Colombia
IMCN-UNSJ	Instituto y Muso de Ciencias Naturales, Universidad Nacional de San Juan	San Juan	Argentina
IMTM	Fundação de Medicina Tropical do Amazonas	Manaus	Brazil
IMZUT	Instituto e Museo di Zoologia della Università di Torino	Torino	Italy
INALI	Instituto Nacioal de Limnologia	Santa Fe	Argentina
INPA	Instituto Nacional de Pesquisas da Amazônia	Manaus	Brazil
IRSBN	Institut Royal des Sciences Naturelles de Belgique	Brussels	Belgium
IVB	Instituto Vital Brazil	Niterói	Brazil
KU	University of Kansas Biodiversity Institute	Lawrence	U.S.A.
LACM	Natural History Museum of Los Angeles Country	Los Angeles	U.S.A.
LGE	Universidad Nacional de Misiones	Misiones	Argentina
LMZ	Universidade Federal de Alfenas	Alfenas	Brazil
LPHA	Linha de Pesquisa em Herpetologia da Amazônia, Laboratório de Pesquisas Zoológicas, Faculdades Integradas do Tapajós	Santarem	Brazil
LSUMZ	Lousiana Museum of Natural History	Baton Rouge	U.S.A.
LZUFOP	Laboratório de Zoologia da Universidade Federal de Ouro Preto	Ouro Preto	Brazil
MACN	Museo Argentino de Ciencias Naturales "Bernadino Rivadavia"	Buenos Aires	Argentina
MBML	Museu de Biologia Prof. Mello Leitão	Santa Teresa	Brazil
MBUCV	Museo de Biologia de la Universidad Central de Venezuela	Caracas	Venezuela
MCN	Museu de Ciencias Naturais da Fundação Zoobotânica do Rio Grande do Sul	Porto Alegre	Brazil

Acronym	Institution	City	Country
MCNG	Museo de Ciencias Naturales da la UNELLEZ	Guanare	Venezuela
MCNR	Museu de Ciências Naturais da Pontifícia Universidade Católica de Minas Gerais	Belo Horizonte	Brazil
MCP	Museu de Ciências e Tecnologia, Pontfícia Universidade Católica do Rio Grande do Sul	Porto Alegre	Brazil
MCZ	Museum of Comparative Zoology	Cambridge	U.K.
MER	Museo de Ciencias Naturales y Antropológicas	Paraná	Argentina
MFA	Museo Provincial de Ciencias Naturales "Florentino Ameghino"	Santa Fé	Argentina
MHNCI	Museu de Historia Natural Capão da Imbuia	Curitiba	Brazil
MHNG	Musém d'Histoire naturelle	Geneve	Switzerland
MHNLS	Museo de Historia Natural La Salle, Fundación La Salle de Ciencias Naturales	Caracas	Venezuela
MHNP	Museu de História Natural, Prefeitura Municipal de Pirassununga	Pirassununga	Brazil
MLP	Museo de la Plata	La Plata	Argentina
MLS	Museo de Historia Natural, Universidad de La Salle	Bogota	Colombia
MNHG	Museum d'Histoire Naturelle	Geneve	France
MNHN	Muséum National D'Histoire naturelle	Paris	France
MNHNM	Museo de Historia Natural y Cultura Ambiental	Chapultepec	Mexico
MNHNP	Museo Nacional de Historia Natural del Paraguay	San Lorenzo	Paraguay
MNKR	Museo de Historia Natural Noel Kempff Mercado	Santa Cruz de La Sierra	Bolivia
MNRJ	Museu Nacional, Universidade Federal do Rio de Janeiro	Rio de Janeiro	Brazil
MPEG	Museu Paraense Emilio Goeldi	Belém	Brazil
MPHNLP	Museo Provincial de Historia Natural de La Pampa	La Pampa	Argentina
MUFAL	Museu da Universidade Federal de Alagoas	Maceió	Brazil
MUSA	Museo de la Universidad de Arequipa	Arequipa	Peru
MUSM	Museo de Historia Natural, Universidad Mayor de San Marcos	Lima	Peru
MVZ	Museum of Vertebrate Zoology	Berkeley	U.S.A.
MZNZ	Museu de Ciências Naturais da Fundação Zoobotanica do Rio Grande do Sul	Porto Alegre	Brazil

Acronym	Institution	City	Country
MZUEFS	Museu de Zoologia da Universidade Estadual de Feira de Santana	Feira de Santana	Brazil
MZUESC	Museu de Zoologia da Universidade Estadual de Santa Cruz	Ilhéus	Brazil
MZUFBA	Museu de Zoologia da Universidade Federal da Bahia	Salvador	Brazil
MZUFV	Museu de Zoologia João Moojen da Universidade Federal de Viçosa	Viçosa	Brazil
MZUSP	Museu de Zoologia da Universidade de São Paulo	São Paulo	Brazil
MZUT	Museo de Zoologia Università di Torino	Turin	Italy
NHMW	Natural History Museum of Vienna	Vienna	Austria
NMG	Naturhistoriska Museum of Göteborg	Göteborg	Sweden
PNEP	Parque Nacional El Pomar	Entre Rios	Argentina
PNLC	Parque Nacional La Campana Reserva de la Bisfera	Marga	Chile
QCAZ	Pontificia Universidad Católica del Ecuador	Quito	Ecuador
RMNH	Natural Biodiversity Center	Leiden	Netherlands
ROM	Royal Ontario Museum, Department of Natural History	Toronto	Canada
SARM	Serpentario Antonio Ruiz de Montoya	Misiones	Argentina
SDSNH	San Diego Society of Natural History, San Diego Natural History Museum	San Diego	U.S.A.
SMF	Senckenberg Museum of Frankfurt	Frankfurt	Germany
SMNS	Staatliches Museum für Naturkunde, Stuttgart, Baden-Württemberg	Stttgart	Germany
SMP	Surinaams Museum; obsolete, transferred to NZCS (National Zoological Collection of Suriname, Anton de Kom University of Suriname)	Paramaribo	Surinam
TCWC	Texas Cooperative Wildlife Collection	Austin	U.S.A.
TU	Tulane University Museum of Natural History	Belle Chase	U.S.A.
UBUNAM-CNAR	Colección Nacional de Anfibios y Reptiles, Universidad Autonoma de Mexico	Mexico City	Mexico
UCICB	Instituto Central de Biologia Universidad de Concepcion	Concepcion	Chile
UCM	University of Colorado Museum of Natural History,	Boulder	U.S.A.
UCR	Universidad de Costa Rica, Escuela de Biología, Museo de Zoología, Departemento de Biología	San José	Costa Rica
UF	University of Florida, Florida Museum of Natural History	Gainesville	U.S.A.

Acronym	Institution	City	Country
UFAC	Universidade Federal do Acre	Rio Branco	Brazil
UFACF	Universidade Federal do Acre, Campus Cruzeiro do Sul	Cruzeiro do Sul	Brazil
UFJF	Universidade Federal de Juiz de Fora	Juiz de Fora	Brazil
UFMG	Universidade Federal de Minas Gerais	Belo Horizonte	Brazil
UFMT	Universidade Federal do Mato Grosso	Cuiabá	Brazil
UFPB	Universidade Federal da Paraíba	João Pessoa	Brazil
UFU	Universidade Federal de Uberlândia	Uberlândia	Brazil
UMMZ	Museum of Zoology, University of Michigan	Ann Arbor	U.S.A.
UNNEC	Universidad Nacional del Nordeste	Corrientes	Argentina
USNM	United States National Museum, Smithsonian Institution	Maryland	U.S.A.
UTA	University of Texas at Arlington	Arlington	U.S.A.
YPM	Yale University, Peabody Museum of Natural History	New Haven	U.S.A.
ZFMK	Zoologisches Forschungsmuseum [formerly Forschungsinstitut und Museum] Alexander Koenig	Bonn	Germany
ZMA	Zoölogisch Museum, Universiteit van Amsterdam	Amsterdam	Netherlands
ZMB	Museum für Naturkunde Berlin	Berlin	Germany
ZMH	Zoological Museum Hamburg [Biozentrum Grindel und Zoologisches Museum; formerly Zoologisches Institut und Museum], Universität Hamburg	Hamburg	Germany
ZMUC	Zoological Museum, University of Copenhagen	Copenhagen	Denmark
ZSMH	Zoologische Staatssammlung München	Munich	Germany
ZUEC	Museu de Zoologia Dr. Adão José Cardoso da Universidade de Campinas	Campinas	Brazil
ZUFMS	Coleção Zoológica de Referência da Universidade Federal de Mato Grosso do Sul	Campo Grande	Brazil
ZUFRJ	Departamento de Zoologia, Instituto de Biologia, Universidade Federal do Rio de Janeiro	Rio de Janeiro	Brazil
ZVC-R	Colección de Zoología Vertebrados de la Facultad de Ciencias, Universidad de la República	Montevideo	Uruguay
