# Supplementary material for: Patterns, biases and prospects in the distribution and diversity of Neotropical snakes
Source: Glob Ecol Biogeogr. 2017 Nov 23;27(1):14–21. doi: 10.1111/geb.12679 (PMC5765514; doi:10.1111/geb.12679)
Supplement: Supplementary file 2 — Supporting Appendix S2 [file GEB-27-14-s002.pdf]

Patterns, biases and prospects in the distribution and diversity of Neotropical snakes

Thaís B. Guedes, Ricardo J. Sawaya, Alexander Zizka, Shawn Laffan, Alexander Pyron, Renato S. Bérnils, Martin Jansen, Paulo Passos, Ana L. C. Prudente, Diego F. Cisneros-Heredia, Henrique B. Braz, Cristiano de C. Nogueira & Alexandre Antonelli

Appendix S2 Database comprising the taxonomic and geographic changes perfomed on Raw Dataset (RD) downloaded from GBIF.

Taxonomic changes

Name provided by GBIF	Updated name
Adelphicos latifasciatus	*Adelphicos latifasciatum*
Adelphicos quadrivirgatus	*Adelphicos quadrivirgatum*
Alsophis anomalus	*Haitiophis anomalus*
Alsophis cantherigerus	*Cubophis cantherigerus*
Alsophis elegans	*Pseudalsophis elegans*
Alsophis portoricensis	*Borikenophis portoricensis*
Alsophis vudii	*Cubophis vudii*
Antillophis andreae	*Caraiba andreae*
Antillophis parvifrons	*Hypsirhynchus parvifrons*
Arrhyton exiguum	*Magliophis exiguum*
Arrhyton landoi	*Arrhyton redimitum*
Bothriopsis bilineata	*Bothrops bilineatus*
Bothriopsis oligolepis	*Bothrops oligolepis*
Bothriopsis punctata	*Bothrops punctatus*
Bothriopsis taeniata	*Bothrops taeniatus*
Cerrophidion barbouri	*Mixcoatlus barbouri*
Clelia bicolor	*Mussurana bicolor*
Conophis pulcher	*Conophis lineatus*
Dendrophidion brunneus	*Dendrophidium brunneum*
Dendrophidion paucicarinatus	*Dendrophidion paucicarinatum*
Dendrophidion percarinatus	*Dendrophidion percarinatum*
Dipsas perijanensis	*Plesiodipsas perijanensis*
Drymobius margaritifera	*Drymobius margaritiferus*
Elapomorphus lepidus	*Coronelaps lepidus*
Epicrates angulifer	*Chilabothrus angulifer*
Epicrates fordii	*Chilabothrus fordii*
Epicrates gracilis	*Chilabothrus gracilis*
Epicrates inornatus	*Chilabothrus inornatus*
Epicrates striatus	*Chilabothrus striatus*
Epictia subcrotillus	*Epictia subcrotilla*
Erythrolamprus bizonus	*Erythrolamprus bizona*
Leptognathus mikani	*Dipsas oreas*
Leptotyphlops albifrons	*Epictia tenella*
Leptotyphlops albipuncta	*Epictia albipuncta*
Leptotyphlops anthracinus	*Trilepida anthracina*

Name provided by GBIF	Updated name
Leptotyphlops australis	*Epictia australis*
Leptotyphlops borrichianus	*Siagonodon borrichianus*
Leptotyphlops cupinensis	*Siagonodon cupinensis*
Leptotyphlops dulcis	*Rena dulcis*
Leptotyphlops goudotii	*Epictia goudotii*
Leptotyphlops humilis	*Rena humilis*
Leptotyphlops joshuai	*Trilepida joshuai*
Leptotyphlops leptepileptus	*Mithophis leptepileptus*
Leptotyphlops macrolepis	*Trilepida macrolepis*
Leptotyphlops maximus	*Rena maxima*
Leptotyphlops melanotermus	*Epictia albipuncta*
Leptotyphlops munoai	*Epictia munoai*
Leptotyphlops pyrites	*Mithophis pyrites*
Leptotyphlops salgueiroi	*Trilepida salgueiroi*
Leptotyphlops subcrotillus	*Epictia subcrotillus*
Leptotyphlops unguirostris	*Rena unguirostris*
Leptotyphlops weyrauchi	*Epictia albipuncta*
Liophis almadensis	*Erythrolamprus almadensis*
Liophis anomalus	*Lygophis anomalus*
Liophis cobellus	*Erythrolamprus cobella*
Liophis dilepis	*Lygophis dilepis*
Liophis epinephelus	*Erythrolamprus epinephelus*
Liophis flavifrenatus	*Lygophis flavifrenatus*
Liophis guentheri	*Erythrolamprus albertguentheri*
Liophis jaegeri	*Erythrolamprus jaegeri*
Liophis lineatus	*Lygophis lineatus*
Liophis melanotus	*Erythrolamprus melanotus*
Liophis miliaris	*Erythrolamprus miliaris*
Liophis poecilogyrus	*Erythrolamprus poecilogyrus*
Liophis reginae	*Erythrolamprus reginae*
Liophis sagittifer	*Erythrolamprus sagittifer*
Liophis taeniurus	*Erythrolamprus taeniurus*
Liophis triscalis	*Erythrolamprus triscalis*
Liophis typhlus	*Erythrolamprus typhlus*
Liophis williamsi	*Erythrolamprus williamsi*
Ophryacus melanurus	*Mixcoatlus melanurus*
Oxyrhopus petola	*Oxyrhopus petolarius*
Pantherophis flavirufus	*Pseudelaphe flavirufa*
Philodryas simonsi	*Philodryas simonsii*
Philodryas varius	*Philodryas varia*
Pseudablabes agassizii	*Philodryas agassizii*
Pseustes poecilonotus	*Phrynonax poecilonotus*
Pseustes shropshirei	*Phrynonax poecilonotus*
Pseustes sulphureus	*Spilotes sulphureus*
Rhadinaea godmani	*Rhadinella godmani*
Rhadinaea hannsteini	*Rhadinella hannsteini*
Rhadinaea hempsteadae	*Rhadinella hempsteadae*
Rhadinaea kanalchutchan	*Rhadinella kanalchutchan*
Rhadinaea kinkelini	*Rhadinella kinkelini*

Name provided by GBIF	Updated name
Rhadinaea lachrymans	*Rhadinella lachrymans*
Sibon annulifera	*Tropidodipsas annulifera*
Sibon dimidiata	*Sibon dimidiatus*
Sibon fischeri	*Tropidodipsas fischeri*
Sibon sanniola	*Sibon sanniolus*
Sistrurus ravus	*Crotalus ravus*
Tantilla cuesta	*Tantilla jani*
Tropidophis melaneura	*Tropidophis melanurus*
Typhlops brongersmianus	*Amerotyphlops brongersmianus*
Typhlops costaricensis	*Amerotyphlops costaricensis*
Typhlops lehneri	*Amerotyphlops lehneri*
Typhlops stadelmani	*Amerotyphlops stadelmani*
Typhlops tasymicris	*Amerotyphlops tasymicris*
Typhlops tenuis	*Amerotyphlops tenuis*
Umbrivaga mertensi	*Erythrolamprus mertensi*
Umbrivaga pygmaea	*Erythrolamprus pygmaeus*
Waglerophis merremi	*Xenodon merremii*
Xenoxybelis argenteus	*Philodryas argentea*
Rhadinaea pilonaorum	*Rhadinella pilonarum*
Rhadinaea montecristi	*Rhadinella montecristi*
Rhadinaea schistosa	*Rhadinella schistosa*
Rhadinaea serperastra	*Rhadinella seperastes*

Geographic changes

Species	Geographical Decision
Bitis arietans	Removed all records, all wrong. It doesn't occur in Neotropical region
Bitis gabonica	Removed all records, all wrong. It doesn't occur in Neotropical region
Bothrops alternatus	Removed one record in the north of the state of Minas Gerais
Bothrops atrox	Removed records in Central America and Southeastern Brazil
Bothrops bilineatus	Removed record in Central America (Guatemala)
Bothrops jararaca	Removed records in Bolivia
Bothrops lanceolatus	Removed records in Atlantic Forest
Calliophis gracilis	Removed all records, all wrong. It doesn't occur in Neotropical region
Chilomeniscus stramineus	Removed all records, all wrong. It doesn't occur in Neotropical region
Chionactis palarostris	Removed all records, all wrong. It doesn't occur in Neotropical region
Chironius bicarinatus	Removed two point records: North of the state of Minas Gerais and one in Goiás
Clelia plumbea	Removed records in Amazonia
Clelia rustica	Removed records in Amazonia
Contia tenuis	Removed all records, all wrong. It doesn't occur in Neotropical region

Species	Geographical Decision
Corallus cookii	Removed all records, all wrong
Crotalus cerastes	Removed all records, all wrong. It doesn't occur in Neotropical region
Crotalus oreganus	Removed all records, all wrong
Crotalus ruber	Removed all records, all wrong
Dendrophidion nuchale	Removed two records in Mexico, Colombia and Ecuador
Dipsas latifrontalis	Removed all records, all wrong. It doesn't occur in Venezuela
Elapomorphus quinquelineatus	Removed one point record between the state of Paraná in Brazil and Paraguay
Erythrolamprus aesculapii	Removed record in Central America (Honduras)
Erythrolamprus almadensis	Removed record in the south of Argentina
Erythrolamprus cobella	Removed records in Peru
Helicops angulatus	Removed one record in the coast of Peru
Helicops carinicaudus	Removed all records, all wrong
Helicops hagmani	Removed all records, all wrong
Helicops leopardinus	Removed all records, all wrong
Lampropeltis getula	Removed all records, all wrong. It doesn't occur in Neotropical region
Lamprophis virgatus	Removed all records, all wrong. It doesn't occur in Neotropical region
Lygophis dilepis	Removed all records, all wrong
Lygophis lineatus	Removed two records in the south of South America
Macroprotodon cucullatus	Removed all records, all wrong. It doesn't occur in Neotropical region
Micrurus corallinus	Removed on record in Goiás
Micrurus frontalis	Removed one point record in Rio Grande do Sul
Micrurus fulvius	Removed all records, all wrong. It doesn't occur in Neotropical region
Micrurus ibiboboca	Removed records in Amazonia
Morelia spilota	Removed all records, all wrong. It doesn't occur in Neotropical region
Nerodia sipedon	Removed all records, all wrong. It doesn't occur in Neotropical region
Nerodia taxispilota	Removed all records, all wrong. It doesn't occur in Neotropical region
Oxyrhopus doliatus	Removed all records, all wrong
Oxyrhopus trigeminus	Removed all records in Paraguay
Pantherophis guttatus	Removed all records, all wrong. It doesn't occur in Neotropical region
Phalotris tricolor	Removed records in Atlantic Forest
Philodryas arnoldoi	Removed records in Atlantic Forest
Philodryas tachymenoides	Removed records in Chile
Pituophis melanoleucus	Removed all records, all wrong. It doesn't occur in Neotropical region
Popeia fucatus	Removed. It's not a valid name for snake species
Psammophis phillipsi	Removed all records, all wrong. It doesn't occur in Neotropical region

Species	Geographical Decision
Ramphotyphlops braminus	Removed all records. This species was introduced by humans in Mexico
Sibynomorphus ventrimaculatus	Removed one record in Argentina
Siphlophis longicaudatus	Removed all records, all wrong
Storeria occipitomaculata	Removed all records, all wrong. It doesn't occur in Neotropical region
Tachymenis affinis	Removed all records, all wrong
Tachymenis peruviana	Removed records in Chile
Tantilla capistrata	Removed all records, all wrong
Tantilla trilineata	Removed all records, all wrong. It doesn't occur in Neotropical region
Thamnodynastes pallidus	Removed all records in the south of Brazil
Thamnophis couchii	Removed all records, all wrong. It doesn't occur in Neotropical region
Thamnophis sauritus	Removed records in Bahamas
Thamnophis sirtalis	Removed all records, all wrong
Xenoxybelis boulengeri	Removed. Nomenclatural uncertainty
