# Supplementary material for: Patterns, biases and prospects in the distribution and diversity of Neotropical snakes
Source: Glob Ecol Biogeogr. 2017 Nov 23;27(1):14–21. doi: 10.1111/geb.12679 (PMC5765514; doi:10.1111/geb.12679)
Supplement: Supplementary file 4 — Supporting Appendix S4 [file GEB-27-14-s004.pdf]

Patterns, biases and prospects in the distribution and diversity of Neotropical snakes

Thaís B. Guedes, Ricardo J. Sawaya, Alexander Zizka, Shawn Laffan, Alexander Pyron, Renato S. Bérnils, Martin Jansen, Paulo Passos, Ana L. C. Prudente, Diego F. Cisneros-Heredia, Henrique B. Braz, Cristiano de C. Nogueira & Alexandre Antonelli

Appendix S4 List of all species and families of Neotropical snakes used in this study and their status of knowledge of geographical distribution. Status of knowledge of geographical distribution: we evaluated, guided by literature and under scrutiny and validation of experts, the geographic distribution reported for each species using the CD. We classified as "poorly-known" (N= 278 spp.) the taxa with few known specimens apparently due to low population density, taxonomic or nomenclature problems, as well as comprising species recently described on the basis of small series (see B criterion in Categories and Criteria 2017-1 in IUCN, see http://www.iucnredlist.org/technical-documents/categories-and-criteria); or "well-known" (N= 608 spp.) the opposite.

Neotropical snakes	Families	Status of knowledge of geographical distribution
Adelophis copei	Natricidae	Poorly-known
Adelphicos latifasciatum	Dipsadidae	Poorly-known
Adelphicos nigrilatum	Dipsadidae	Well-known
Adelphicos quadrivirgatum	Dipsadidae	Well-known
Adelphicos veraepacis	Dipsadidae	Poorly-known
Agkistrodon bilineatus	Viperidae	Well-known
Alsophis rijgersmaei	Dipsadidae	Well-known
Alsophis rufiventris	Dipsadidae	Well-known
Amastridium veliferum	Dipsadidae	Poorly-known
Amerotyphlops amoipira	Typhlopidae	Well-known
Amerotyphlops arenensis	Typhlopidae	Poorly-known
Amerotyphlops brongersmianus	Typhlopidae	Well-known
Amerotyphlops costaricensis	Typhlopidae	Poorly-known
Amerotyphlops lehneri	Typhlopidae	Poorly-known

Neotropical snakes	Families	Status of knowledge of geographical distribution
Amerotyphlops minuisquamus	Typhlopidae	Well-known
Amerotyphlops paucisquamus	Typhlopidae	Well-known
Amerotyphlops reticulatus	Typhlopidae	Well-known
Amerotyphlops stadelmani	Typhlopidae	Poorly-known
Amerotyphlops tasymicris	Typhlopidae	Well-known
Amerotyphlops tenuis	Typhlopidae	Poorly-known
Amerotyphlops yonenagae	Typhlopidae	Poorly-known
Anilius scytale	Aniliidae	Well-known
Apostolepis aff. *pymi*	Dipsadidae	Well-known
Apostolepis albicollaris	Dipsadidae	Well-known
Apostolepis ambinigra	Dipsadidae	Well-known
Apostolepis ammodites	Dipsadidae	Well-known
Apostolepis arenaria	Dipsadidae	Poorly-known
Apostolepis assimilis	Dipsadidae	Well-known
Apostolepis cearensis	Dipsadidae	Well-known
Apostolepis cerradoensis	Dipsadidae	Poorly-known
Apostolepis christineae	Dipsadidae	Poorly-known
Apostolepis dimidiata	Dipsadidae	Well-known
Apostolepis dorbignyi	Dipsadidae	Poorly-known
Apostolepis flavotorquata	Dipsadidae	Well-known
Apostolepis gaboi	Dipsadidae	Well-known
Apostolepis goiasensis	Dipsadidae	Well-known
Apostolepis intermedia	Dipsadidae	Well-known
Apostolepis lineata	Dipsadidae	Poorly-known
Apostolepis longicaudata	Dipsadidae	Well-known
Apostolepis multicincta	Dipsadidae	Well-known
Apostolepis nelsonjorgei	Dipsadidae	Well-known

Neotropical snakes	Families	Status of knowledge of geographical distribution
Apostolepis niceforoi	Dipsadidae	Poorly-known
Apostolepis nigrolineata	Dipsadidae	Well-known
Apostolepis nigroterminata	Dipsadidae	Well-known
Apostolepis phillipsi	Dipsadidae	Poorly-known
Apostolepis polylepis	Dipsadidae	Well-known
Apostolepis pymi	Dipsadidae	Well-known
Apostolepis quirogai	Dipsadidae	Poorly-known
Apostolepis serrana	Dipsadidae	Poorly-known
Apostolepis striata	Dipsadidae	Poorly-known
Apostolepis tertulianobeui	Dipsadidae	Well-known
Apostolepis vittata	Dipsadidae	Poorly-known
Arizona elegans	Colubridae	Poorly-known
Arrhyton dolichura	Dipsadidae	Well-known
Arrhyton redimitum	Dipsadidae	Well-known
Arrhyton taeniatum	Dipsadidae	Well-known
Arrhyton vittatum	Dipsadidae	Well-known
Atractus albuquerquei	Dipsadidae	Well-known
Atractus alphonsehogei	Dipsadidae	Well-known
Atractus altagratiae	Dipsadidae	Poorly-known
Atractus badius	Dipsadidae	Well-known
Atractus bocki	Dipsadidae	Poorly-known
Atractus bocourti	Dipsadidae	Poorly-known
Atractus boettgeri	Dipsadidae	Poorly-known
Atractus caete	Dipsadidae	Poorly-known
Atractus carrioni	Dipsadidae	Poorly-known
Atractus caxiuana	Dipsadidae	Well-known
Atractus charitoae	Dipsadidae	Poorly-known

Neotropical snakes	Families	Status of knowledge of geographical distribution
Atractus collaris	Dipsadidae	Well-known
Atractus crassicaudatus	Dipsadidae	Poorly-known
Atractus dunni	Dipsadidae	Poorly-known
Atractus edioi	Dipsadidae	Poorly-known
Atractus elaps	Dipsadidae	Well-known
Atractus emmeli	Dipsadidae	Well-known
Atractus erythromelas	Dipsadidae	Poorly-known
Atractus flammigerus	Dipsadidae	Poorly-known
Atractus franciscopaivai	Dipsadidae	Poorly-known
Atractus francoi	Dipsadidae	Well-known
Atractus fuliginosus	Dipsadidae	Poorly-known
Atractus gaigeae	Dipsadidae	Poorly-known
Atractus guentheri	Dipsadidae	Well-known
Atractus heliobelluomini	Dipsadidae	Poorly-known
Atractus hoogmoedi	Dipsadidae	Poorly-known
Atractus insipidus	Dipsadidae	Poorly-known
Atractus latifrons	Dipsadidae	Well-known
Atractus lehmanni	Dipsadidae	Poorly-known
Atractus maculatus	Dipsadidae	Well-known
Atractus major	Dipsadidae	Well-known
Atractus microrhynchus	Dipsadidae	Poorly-known
Atractus multicinctus	Dipsadidae	Poorly-known
Atractus natans	Dipsadidae	Well-known
Atractus obtusirostris	Dipsadidae	Poorly-known
Atractus occidentalis	Dipsadidae	Poorly-known
Atractus occipitoalbus	Dipsadidae	Poorly-known
Atractus oculotemporalis	Dipsadidae	Poorly-known

Neotropical snakes	Families	Status of knowledge of geographical distribution
Atractus orcesi	Dipsadidae	Poorly-known
Atractus pantostictus	Dipsadidae	Well-known
Atractus paraguayensis	Dipsadidae	Well-known
Atractus paucidens	Dipsadidae	Well-known
Atractus poeppigi	Dipsadidae	Well-known
Atractus potschi	Dipsadidae	Well-known
Atractus punctiventris	Dipsadidae	Poorly-known
Atractus reticulatus	Dipsadidae	Well-known
Atractus riveroi	Dipsadidae	Poorly-known
Atractus ronnie	Dipsadidae	Well-known
Atractus roulei	Dipsadidae	Poorly-known
Atractus sanctaemartae	Dipsadidae	Poorly-known
Atractus schach	Dipsadidae	Well-known
Atractus serranus	Dipsadidae	Well-known
Atractus snethlageae	Dipsadidae	Well-known
Atractus spinalis	Dipsadidae	Poorly-known
Atractus surucucu	Dipsadidae	Poorly-known
Atractus thalesdelemai	Dipsadidae	Poorly-known
Atractus torquatus	Dipsadidae	Well-known
Atractus trihedrurus	Dipsadidae	Well-known
Atractus trilineatus	Dipsadidae	Well-known
Atractus univittatus	Dipsadidae	Poorly-known
Atractus ventrimaculatus	Dipsadidae	Poorly-known
Atractus zebrinus	Dipsadidae	Well-known
Atractus zidoki	Dipsadidae	Well-known
Atropoides nummifer	Viperidae	Well-known
Atropoides picadoi	Viperidae	Well-known

Neotropical snakes	Families	Status of knowledge of geographical distribution
Boa constrictor	Boidae	Well-known
Boiruna maculata	Dipsadidae	Well-known
Boiruna sertaneja	Dipsadidae	Well-known
Borikenophis portoricensis	Dipsadidae	Well-known
Bothriechis aurifer	Viperidae	Poorly-known
Bothriechis bicolor	Viperidae	Poorly-known
Bothriechis lateralis	Viperidae	Well-known
Bothriechis marchi	Viperidae	Poorly-known
Bothriechis nigroviridis	Viperidae	Well-known
Bothriechis rowleyi	Viperidae	Poorly-known
Bothriechis schlegelii	Viperidae	Poorly-known
Bothriechis thalassinus	Viperidae	Poorly-known
Bothrocophias hyoprora	Viperidae	Well-known
Bothrocophias microphthalmus	Viperidae	Well-known
Bothrocophias myersi	Viperidae	Poorly-known
Bothrops alcatraz	Viperidae	Well-known
Bothrops alternatus	Viperidae	Well-known
Bothrops ammodytoides	Viperidae	Well-known
Bothrops andianus	Viperidae	Poorly-known
Bothrops asper	Viperidae	Well-known
Bothrops atrox	Viperidae	Well-known
Bothrops barnetti	Viperidae	Poorly-known
Bothrops bilineatus	Viperidae	Well-known
Bothrops brazili	Viperidae	Well-known
Bothrops cotiara	Viperidae	Well-known
Bothrops diporus	Viperidae	Well-known
Bothrops erythromelas	Viperidae	Well-known

Neotropical snakes	Families	Status of knowledge of geographical distribution
Bothrops fonsecai	Viperidae	Well-known
Bothrops insularis	Viperidae	Well-known
Bothrops itapetiningae	Viperidae	Well-known
Bothrops jararaca	Viperidae	Well-known
Bothrops jararacussu	Viperidae	Well-known
Bothrops leucurus	Viperidae	Well-known
Bothrops lojanus	Viperidae	Poorly-known
Bothrops lutzi	Viperidae	Well-known
Bothrops marajoensis	Viperidae	Well-known
Bothrops marmoratus	Viperidae	Well-known
Bothrops mattogrossensis	Viperidae	Well-known
Bothrops moojeni	Viperidae	Well-known
Bothrops muriciensis	Viperidae	Poorly-known
Bothrops neuwiedi	Viperidae	Well-known
Bothrops oligolepis	Viperidae	Poorly-known
Bothrops otavioi	Viperidae	Well-known
Bothrops pauloensis	Viperidae	Well-known
Bothrops pictus	Viperidae	Poorly-known
Bothrops pirajai	Viperidae	Well-known
Bothrops pubescens	Viperidae	Well-known
Bothrops pulchra	Viperidae	Poorly-known
Bothrops punctatus	Viperidae	Poorly-known
Bothrops taeniatus	Viperidae	Well-known
Bothrops venezuelensis	Viperidae	Poorly-known
Caaeteboia amarali	Dipsadidae	Well-known
Calamodontophis paucidens	Dipsadidae	Well-known
Calamodontophis ronaldoi	Dipsadidae	Poorly-known

Neotropical snakes	Families	Status of knowledge of geographical distribution
Caraiba andreae	Dipsadidae	Well-known
Cerrophidion godmani	Viperidae	Well-known
Cerrophidion tzotzilorum	Viperidae	Poorly-known
Chapinophis xanthocheilus	Dipsadidae	Poorly-known
Chersodromus liebmanni	Dipsadidae	Poorly-known
Chilabothrus angulifer	Boidae	Well-known
Chilabothrus fordii	Boidae	Well-known
Chilabothrus gracilis	Boidae	Well-known
Chilabothrus inornatus	Boidae	Well-known
Chilabothrus striatus	Boidae	Well-known
Chironius bicarinatus	Colubridae	Well-known
Chironius brazili	Colubridae	Well-known
Chironius carinatus	Colubridae	Well-known
Chironius diamantina	Colubridae	Well-known
Chironius exoletus	Colubridae	Well-known
Chironius flavolineatus	Colubridae	Well-known
Chironius flavopictus	Colubridae	Poorly-known
Chironius foveatus	Colubridae	Well-known
Chironius fuscus	Colubridae	Well-known
Chironius grandisquamis	Colubridae	Poorly-known
Chironius laevicollis	Colubridae	Well-known
Chironius laurenti	Colubridae	Well-known
Chironius maculoventris	Colubridae	Well-known
Chironius monticola	Colubridae	Poorly-known
Chironius multiventris	Colubridae	Well-known
Chironius quadricarinatus	Colubridae	Well-known
Chironius scurrulus	Colubridae	Well-known

**Neotropical snakes**	**Families**	**Status of knowledge of geographical distribution**
Chironius septentrionalis	Colubridae	Well-known
Clelia clelia	Dipsadidae	Well-known
Clelia equatoriana	Dipsadidae	Well-known
Clelia errabunda	Dipsadidae	Well-known
Clelia hussami	Dipsadidae	Well-known
Clelia langeri	Dipsadidae	Well-known
Clelia plumbea	Dipsadidae	Well-known
Clelia scytalina	Dipsadidae	Well-known
Coluber bilineatus	Colubridae	Well-known
Coluber constrictor	Colubridae	Well-known
Coluber flagellum	Colubridae	Poorly-known
Coluber mentovarius	Colubridae	Well-known
Coluber schotti	Colubridae	Well-known
Coluber taeniatus	Colubridae	Well-known
Coniophanes alvarezi	Dipsadidae	Well-known
Coniophanes bipunctatus	Dipsadidae	Poorly-known
Coniophanes fissidens	Dipsadidae	Well-known
Coniophanes imperialis	Dipsadidae	Well-known
Coniophanes lateritius	Dipsadidae	Well-known
Coniophanes meridanus	Dipsadidae	Poorly-known
Coniophanes piceivittis	Dipsadidae	Well-known
Coniophanes schmidti	Dipsadidae	Well-known
Conophis lineatus	Dipsadidae	Well-known
Conophis vittatus	Dipsadidae	Well-known
Conopsis biserialis	Colubridae	Well-known
Conopsis lineata	Colubridae	Well-known
Conopsis megalodon	Colubridae	Well-known

Neotropical snakes	Families	Status of knowledge of geographical distribution
Conopsis nasus	Colubridae	Well-known
Corallus annulatus	Boidae	Poorly-known
Corallus batesii	Boidae	Well-known
Corallus caninus	Boidae	Well-known
Corallus cookii	Boidae	Well-known
Corallus cropanii	Boidae	Poorly-known
Corallus hortulanus	Boidae	Well-known
Corallus ruschenbergerii	Boidae	Poorly-known
Coronelaps lepidus	Dipsadidae	Well-known
Crisantophis nevermanni	Dipsadidae	Poorly-known
Crotalus aquilus	Viperidae	Well-known
Crotalus atrox	Viperidae	Well-known
Crotalus basiliscus	Viperidae	Well-known
Crotalus durissus	Viperidae	Well-known
Crotalus intermedius	Viperidae	Well-known
Crotalus lepidus	Viperidae	Poorly-known
Crotalus molossus	Viperidae	Well-known
Crotalus polystictus	Viperidae	Well-known
Crotalus pricei	Viperidae	Poorly-known
Crotalus pusillus	Viperidae	Well-known
Crotalus ravus	Viperidae	Well-known
Crotalus scutulatus	Viperidae	Poorly-known
Crotalus stejnegeri	Viperidae	Poorly-known
Crotalus tigris	Viperidae	Poorly-known
Crotalus triseriatus	Viperidae	Well-known
Crotalus willardi	Viperidae	Poorly-known
Cryophis hallbergi	Dipsadidae	Poorly-known

Neotropical snakes	Families	Status of knowledge of geographical distribution
Cubophis cantherigerus	Dipsadidae	Well-known
Cubophis vudii	Dipsadidae	Well-known
Dendrophidion atlantica	Colubridae	Well-known
Dendrophidion bivittatum	Colubridae	Poorly-known
Dendrophidion brunneum	Colubridae	Poorly-known
Dendrophidion dendrophis	Colubridae	Well-known
Dendrophidion nuchale	Colubridae	Poorly-known
Dendrophidion paucicarinatum	Colubridae	Well-known
Dendrophidion percarinatum	Colubridae	Poorly-known
Dendrophidion vinitor	Colubridae	Well-known
Diadophis punctatus	Dipsadidae	Poorly-known
Dipsas albifrons	Dipsadidae	Well-known
Dipsas alternans	Dipsadidae	Well-known
Dipsas andiana	Dipsadidae	Well-known
Dipsas articulata	Dipsadidae	Poorly-known
Dipsas brevifacies	Dipsadidae	Well-known
Dipsas bucephala	Dipsadidae	Well-known
Dipsas catesbyi	Dipsadidae	Well-known
Dipsas copei	Dipsadidae	Well-known
Dipsas gracilis	Dipsadidae	Poorly-known
Dipsas indica	Dipsadidae	Well-known
Dipsas oreas	Dipsadidae	Well-known
Dipsas pavonina	Dipsadidae	Well-known
Dipsas peruana	Dipsadidae	Poorly-known
Dipsas sanctijoannis	Dipsadidae	Poorly-known
Dipsas sazimai	Dipsadidae	Well-known
Dipsas variegata	Dipsadidae	Well-known

Neotropical snakes	Families	Status of knowledge of geographical distribution
Dipsas vermiculata	Dipsadidae	Poorly-known
Ditaxodon taeniatus	Dipsadidae	Well-known
Drepanoides anomalus	Dipsadidae	Well-known
Drymarchon corais	Colubridae	Well-known
Drymarchon melanurus	Colubridae	Poorly-known
Drymobius chloroticus	Colubridae	Well-known
Drymobius margaritiferus	Colubridae	Well-known
Drymobius melanotropis	Colubridae	Poorly-known
Drymobius rhombifer	Colubridae	Well-known
Drymoluber brazili	Colubridae	Well-known
Drymoluber dichrous	Colubridae	Well-known
Echinanthera amoena	Dipsadidae	Well-known
Echinanthera cephalomaculata	Dipsadidae	Poorly-known
Echinanthera cephalostriata	Dipsadidae	Well-known
Echinanthera cyanopleura	Dipsadidae	Well-known
Echinanthera melanostigma	Dipsadidae	Well-known
Echinanthera undulata	Dipsadidae	Well-known
Elapomorphus quinquelineatus	Dipsadidae	Well-known
Elapomorphus wuchereri	Dipsadidae	Well-known
Enuliophis sclateri	Dipsadidae	Poorly-known
Enulius flavitorques	Dipsadidae	Well-known
Enulius oligostichus	Dipsadidae	Poorly-known
Epicrates alvarezi	Boidae	Well-known
Epicrates assisi	Boidae	Well-known
Epicrates cenchria	Boidae	Well-known
Epicrates crassus	Boidae	Well-known
Epicrates maurus	Boidae	Well-known

Neotropical snakes	Families	Status of knowledge of geographical distribution
Epictia albipuncta	Leptotyphlopidae	Well-known
Epictia australis	Leptotyphlopidae	Well-known
Epictia borapeliotes	Leptotyphlopidae	Well-known
Epictia clinorostris	Leptotyphlopidae	Poorly-known
Epictia diaplocia	Leptotyphlopidae	Poorly-known
Epictia goudotii	Leptotyphlopidae	Well-known
Epictia munoai	Leptotyphlopidae	Well-known
Epictia striatula	Leptotyphlopidae	Well-known
Epictia subcrotilla	Leptotyphlopidae	Poorly-known
Epictia tenella	Leptotyphlopidae	Well-known
Epictia vellardi	Leptotyphlopidae	Well-known
Erythrolamprus aesculapii	Dipsadidae	Well-known
Erythrolamprus albertguentheri	Dipsadidae	Well-known
Erythrolamprus almadensis	Dipsadidae	Well-known
Erythrolamprus atraventer	Dipsadidae	Well-known
Erythrolamprus bizona	Dipsadidae	Well-known
Erythrolamprus breviceps	Dipsadidae	Well-known
Erythrolamprus carajasensis	Dipsadidae	Well-known
Erythrolamprus ceii	Dipsadidae	Poorly-known
Erythrolamprus cobella	Dipsadidae	Well-known
Erythrolamprus dorsocorallinus	Dipsadidae	Well-known
Erythrolamprus epinephelus	Dipsadidae	Well-known
Erythrolamprus frenatus	Dipsadidae	Well-known
Erythrolamprus jaegeri	Dipsadidae	Well-known
Erythrolamprus maryellenae	Dipsadidae	Well-known
Erythrolamprus melanotus	Dipsadidae	Well-known
Erythrolamprus mertensi	Dipsadidae	Poorly-known

Neotropical snakes	Families	Status of knowledge of geographical distribution
Erythrolamprus miliaris	Dipsadidae	Well-known
Erythrolamprus mimus	Dipsadidae	Well-known
Erythrolamprus mossoroensis	Dipsadidae	Well-known
Erythrolamprus oligolepis	Dipsadidae	Well-known
Erythrolamprus poecilogyrus	Dipsadidae	Well-known
Erythrolamprus pygmaeus	Dipsadidae	Well-known
Erythrolamprus reginae	Dipsadidae	Well-known
Erythrolamprus sagittifer	Dipsadidae	Well-known
Erythrolamprus semiaureus	Dipsadidae	Well-known
Erythrolamprus taeniogaster	Dipsadidae	Well-known
Erythrolamprus taeniurus	Dipsadidae	Poorly-known
Erythrolamprus triscalis	Dipsadidae	Well-known
Erythrolamprus typhlus	Dipsadidae	Well-known
Erythrolamprus viridis	Dipsadidae	Well-known
Erythrolamprus williamsi	Dipsadidae	Poorly-known
Erythrolamprus zweifeli	Dipsadidae	Well-known
Eunectes deschauenseei	Boidae	Well-known
Eunectes murinus	Boidae	Well-known
Eunectes notaeus	Boidae	Well-known
Exiliboa placata	Boidae	Well-known
Ficimia hardyi	Colubridae	Poorly-known
Ficimia olivacea	Colubridae	Well-known
Ficimia publia	Colubridae	Well-known
Ficimia streckeri	Colubridae	Poorly-known
Ficimia variegata	Colubridae	Poorly-known
Geagras redimitus	Colubridae	Well-known
Geophis anocularis	Dipsadidae	Poorly-known

Neotropical snakes	Families	Status of knowledge of geographical distribution
Geophis betaniensis	Dipsadidae	Poorly-known
Geophis bicolor	Dipsadidae	Poorly-known
Geophis brachycephalus	Dipsadidae	Well-known
Geophis cancellatus	Dipsadidae	Well-known
Geophis championi	Dipsadidae	Poorly-known
Geophis downsi	Dipsadidae	Poorly-known
Geophis dubius	Dipsadidae	Poorly-known
Geophis dugesii	Dipsadidae	Poorly-known
Geophis fulvoguttatus	Dipsadidae	Poorly-known
Geophis godmani	Dipsadidae	Well-known
Geophis hoffmanni	Dipsadidae	Poorly-known
Geophis immaculatus	Dipsadidae	Well-known
Geophis laticinctus	Dipsadidae	Poorly-known
Geophis latifrontalis	Dipsadidae	Well-known
Geophis mutitorques	Dipsadidae	Well-known
Geophis nasalis	Dipsadidae	Well-known
Geophis nigroalbus	Dipsadidae	Poorly-known
Geophis omiltemanus	Dipsadidae	Poorly-known
Geophis rhodogaster	Dipsadidae	Well-known
Geophis ruthveni	Dipsadidae	Well-known
Geophis sallaei	Dipsadidae	Poorly-known
Geophis semidoliatus	Dipsadidae	Well-known
Geophis talamancae	Dipsadidae	Well-known
Geophis tarascae	Dipsadidae	Poorly-known
Geophis zeledoni	Dipsadidae	Well-known
Gomesophis brasiliensis	Dipsadidae	Well-known
Gyalopion canum	Colubridae	Poorly-known

Neotropical snakes	Families	Status of knowledge of geographical distribution
Gyalopion quadrangulare	Colubridae	Well-known
Haitiophis anomalus	Dipsadidae	Well-known
Helicops angulatus	Dipsadidae	Well-known
Helicops apiaka	Dipsadidae	Well-known
Helicops carinicaudus	Dipsadidae	Well-known
Helicops danieli	Dipsadidae	Well-known
Helicops gomesi	Dipsadidae	Well-known
Helicops hagmanni	Dipsadidae	Well-known
Helicops infrataeniatus	Dipsadidae	Well-known
Helicops leopardinus	Dipsadidae	Well-known
Helicops modestus	Dipsadidae	Well-known
Helicops pastazae	Dipsadidae	Well-known
Helicops petersi	Dipsadidae	Well-known
Helicops polylepis	Dipsadidae	Well-known
Helicops scalaris	Dipsadidae	Well-known
Helicops tapajonicus	Dipsadidae	Well-known
Helicops trivittatus	Dipsadidae	Well-known
Hydrodynastes bicinctus	Dipsadidae	Well-known
Hydrodynastes gigas	Dipsadidae	Well-known
Hydrodynastes melanogigas	Dipsadidae	Well-known
Hydromorphus concolor	Dipsadidae	Well-known
Hydromorphus dunni	Dipsadidae	Poorly-known
Hydrops caesurus	Dipsadidae	Well-known
Hydrops martii	Dipsadidae	Well-known
Hydrops triangularis	Dipsadidae	Well-known
Hypsiglena tanzeri	Dipsadidae	Poorly-known
Hypsiglena torquata	Dipsadidae	Well-known

Neotropical snakes	Families	Status of knowledge of geographical distribution
Hypsirhynchus ferox	Dipsadidae	Well-known
Hypsirhynchus parvifrons	Dipsadidae	Well-known
Ialtris agyrtes	Dipsadidae	Well-known
Ialtris dorsalis	Dipsadidae	Well-known
Ialtris haetianus	Dipsadidae	Well-known
Ialtris parishi	Dipsadidae	Well-known
Imantodes cenchoa	Dipsadidae	Well-known
Imantodes gemmistratus	Dipsadidae	Well-known
Imantodes inornatus	Dipsadidae	Poorly-known
Imantodes lentiferus	Dipsadidae	Well-known
Imantodes tenuissimus	Dipsadidae	Well-known
Lachesis melanocephala	Viperidae	Well-known
Lachesis muta	Viperidae	Well-known
Lachesis stenophrys	Viperidae	Well-known
Lampropeltis mexicana	Colubridae	Poorly-known
Lampropeltis pyromelana	Colubridae	Poorly-known
Lampropeltis ruthveni	Colubridae	Poorly-known
Lampropeltis triangulum	Colubridae	Well-known
Leptodeira annulata	Dipsadidae	Well-known
Leptodeira frenata	Dipsadidae	Well-known
Leptodeira maculata	Dipsadidae	Well-known
Leptodeira nigrofasciata	Dipsadidae	Well-known
Leptodeira punctata	Dipsadidae	Well-known
Leptodeira rubricata	Dipsadidae	Poorly-known
Leptodeira septentrionalis	Dipsadidae	Well-known
Leptodeira splendida	Dipsadidae	Well-known
Leptodrymus pulcherrimus	Colubridae	Well-known

Neotropical snakes	Families	Status of knowledge of geographical distribution
Leptophis ahaetulla	Colubridae	Well-known
Leptophis cupreus	Colubridae	Poorly-known
Leptophis depressirostris	Colubridae	Poorly-known
Leptophis diplotropis	Colubridae	Well-known
Leptophis mexicanus	Colubridae	Well-known
Leptophis modestus	Colubridae	Poorly-known
Leptophis nebulosus	Colubridae	Poorly-known
Leptophis riveti	Colubridae	Poorly-known
Lioheterophis iheringi	Dipsadidae	Poorly-known
Liotyphlops beui	Anomalepididae	Well-known
Liotyphlops caissara	Anomalepididae	Poorly-known
Liotyphlops schubarti	Anomalepididae	Poorly-known
Liotyphlops ternetzii	Anomalepididae	Well-known
Liotyphlops trefauti	Anomalepididae	Poorly-known
Liotyphlops wilderi	Anomalepididae	Well-known
Loxocemus bicolor	Loxocemidae	Well-known
Lygophis anomalus	Dipsadidae	Well-known
Lygophis dilepis	Dipsadidae	Well-known
Lygophis flavifrenatus	Dipsadidae	Well-known
Lygophis lineatus	Dipsadidae	Well-known
Lygophis meridionalis	Dipsadidae	Well-known
Lygophis paucidens	Dipsadidae	Well-known
Magliophis exiguum	Dipsadidae	Well-known
Manolepis putnami	Dipsadidae	Well-known
Mastigodryas amarali	Colubridae	Poorly-known
Mastigodryas bifossatus	Colubridae	Well-known
Mastigodryas boddaerti	Colubridae	Well-known

**Neotropical snakes**	**Families**	**Status of knowledge of geographical distribution**
Mastigodryas cliftoni	Colubridae	Well-known
Mastigodryas danieli	Colubridae	Poorly-known
Mastigodryas heathii	Colubridae	Poorly-known
Mastigodryas melanolomus	Colubridae	Well-known
Mastigodryas moratoi	Colubridae	Well-known
Mastigodryas pleei	Colubridae	Well-known
Mastigodryas pulchriceps	Colubridae	Poorly-known
Mastigodryas reticulatus	Colubridae	Poorly-known
Micruroides euryxanthus	Elapidae	Poorly-known
Micrurus albicinctus	Elapidae	Well-known
Micrurus alleni	Elapidae	Well-known
Micrurus altirostris	Elapidae	Well-known
Micrurus ancoralis	Elapidae	Poorly-known
Micrurus annellatus	Elapidae	Well-known
Micrurus averyi	Elapidae	Well-known
Micrurus baliocoryphus	Elapidae	Well-known
Micrurus bocourti	Elapidae	Poorly-known
Micrurus brasiliensis	Elapidae	Well-known
Micrurus browni	Elapidae	Well-known
Micrurus clarki	Elapidae	Poorly-known
Micrurus collaris	Elapidae	Well-known
Micrurus corallinus	Elapidae	Well-known
Micrurus decoratus	Elapidae	Well-known
Micrurus diana	Elapidae	Well-known
Micrurus diastema	Elapidae	Well-known
Micrurus distans	Elapidae	Well-known
Micrurus dumerilii	Elapidae	Poorly-known

Neotropical snakes	Families	Status of knowledge of geographical distribution
Micrurus elegans	Elapidae	Well-known
Micrurus ephippifer	Elapidae	Well-known
Micrurus filiformis	Elapidae	Well-known
Micrurus frontalis	Elapidae	Well-known
Micrurus hemprichii	Elapidae	Well-known
Micrurus ibiboboca	Elapidae	Well-known
Micrurus isozonus	Elapidae	Well-known
Micrurus langsdorffi	Elapidae	Well-known
Micrurus laticollaris	Elapidae	Poorly-known
Micrurus latifasciatus	Elapidae	Poorly-known
Micrurus lemniscatus	Elapidae	Well-known
Micrurus mertensi	Elapidae	Poorly-known
Micrurus mipartitus	Elapidae	Well-known
Micrurus narduccii	Elapidae	Well-known
Micrurus nattereri	Elapidae	Well-known
Micrurus nigrocinctus	Elapidae	Well-known
Micrurus obscurus	Elapidae	Poorly-known
Micrurus ornatissimus	Elapidae	Poorly-known
Micrurus pacaraimae	Elapidae	Poorly-known
Micrurus paraensis	Elapidae	Well-known
Micrurus peruvianus	Elapidae	Poorly-known
Micrurus potyguara	Elapidae	Well-known
Micrurus proximans	Elapidae	Poorly-known
Micrurus psyches	Elapidae	Well-known
Micrurus putumayensis	Elapidae	Poorly-known
Micrurus pyrrhocryptus	Elapidae	Well-known
Micrurus remotus	Elapidae	Poorly-known

Neotropical snakes	Families	Status of knowledge of geographical distribution
Micrurus ruatanus	Elapidae	Well-known
Micrurus scutiventris	Elapidae	Well-known
Micrurus serranus	Elapidae	Well-known
Micrurus silviae	Elapidae	Well-known
Micrurus spixii	Elapidae	Well-known
Micrurus steindachneri	Elapidae	Poorly-known
Micrurus surinamensis	Elapidae	Well-known
Micrurus tschudii	Elapidae	Poorly-known
Mitophis leptipileptus	Leptotyphlopidae	Well-known
Mitophis pyrites	Leptotyphlopidae	Well-known
Mixcoatlus barbouri	Viperidae	Poorly-known
Mixcoatlus melanurus	Viperidae	Poorly-known
Mussurana bicolor	Dipsadidae	Well-known
Mussurana montana	Dipsadidae	Well-known
Mussurana quimi	Dipsadidae	Well-known
Nerodia rhombifer	Natricidae	Poorly-known
Ninia atrata	Dipsadidae	Well-known
Ninia celata	Dipsadidae	Poorly-known
Ninia diademata	Dipsadidae	Well-known
Ninia espinali	Dipsadidae	Poorly-known
Ninia hudsoni	Dipsadidae	Well-known
Ninia maculata	Dipsadidae	Well-known
Ninia psephota	Dipsadidae	Well-known
Ninia sebae	Dipsadidae	Well-known
Nothopsis rugosus	Dipsadidae	Poorly-known
Opheodrys aestivus	Colubridae	Poorly-known
Oxybelis aeneus	Colubridae	Well-known

Neotropical snakes	Families	Status of knowledge of geographical distribution
Oxybelis brevirostris	Colubridae	Well-known
Oxybelis fulgidus	Colubridae	Well-known
Oxybelis wilsoni	Colubridae	Well-known
Oxyrhopus clathratus	Dipsadidae	Well-known
Oxyrhopus doliatus	Dipsadidae	Poorly-known
Oxyrhopus fitzingeri	Dipsadidae	Poorly-known
Oxyrhopus formosus	Dipsadidae	Well-known
Oxyrhopus guibei	Dipsadidae	Well-known
Oxyrhopus leucomelas	Dipsadidae	Poorly-known
Oxyrhopus marcapatae	Dipsadidae	Poorly-known
Oxyrhopus melanogenys	Dipsadidae	Well-known
Oxyrhopus occipitalis	Dipsadidae	Well-known
Oxyrhopus petolarius	Dipsadidae	Well-known
Oxyrhopus rhombifer	Dipsadidae	Well-known
Oxyrhopus sp.	Dipsadidae	Well-known
Oxyrhopus trigeminus	Dipsadidae	Well-known
Oxyrhopus vanidicus	Dipsadidae	Well-known
Pantherophis emoryi	Colubridae	Poorly-known
Paraphimophis rusticus	Dipsadidae	Well-known
Phalotris bilineatus	Dipsadidae	Well-known
Phalotris concolor	Dipsadidae	Well-known
Phalotris cuyanus	Dipsadidae	Poorly-known
Phalotris labiomaculatus	Dipsadidae	Well-known
Phalotris lativittatus	Dipsadidae	Well-known
Phalotris lemniscatus	Dipsadidae	Well-known
Phalotris matogrossensis	Dipsadidae	Well-known
Phalotris mertensi	Dipsadidae	Well-known

**Neotropical snakes**	**Families**	**Status of knowledge of geographical distribution**
Phalotris multipunctatus	Dipsadidae	Poorly-known
Phalotris nasutus	Dipsadidae	Well-known
Phalotris nigrilatus	Dipsadidae	Well-known
Phalotris reticulatus	Dipsadidae	Poorly-known
Phalotris sansebastiani	Dipsadidae	Poorly-known
Phalotris tricolor	Dipsadidae	Well-known
Philodryas aestiva	Dipsadidae	Well-known
Philodryas agassizii	Dipsadidae	Well-known
Philodryas argentea	Dipsadidae	Well-known
Philodryas arnaldoi	Dipsadidae	Well-known
Philodryas baroni	Dipsadidae	Well-known
Philodryas boliviana	Dipsadidae	Poorly-known
Philodryas chamissonis	Dipsadidae	Well-known
Philodryas georgeboulengeri	Dipsadidae	Well-known
Philodryas laticeps	Dipsadidae	Well-known
Philodryas livida	Dipsadidae	Well-known
Philodryas mattogrossensis	Dipsadidae	Well-known
Philodryas nattereri	Dipsadidae	Well-known
Philodryas olfersii	Dipsadidae	Well-known
Philodryas patagoniensis	Dipsadidae	Well-known
Philodryas psammophidea	Dipsadidae	Well-known
Philodryas simonsii	Dipsadidae	Poorly-known
Philodryas tachymenoides	Dipsadidae	Poorly-known
Philodryas trilineata	Dipsadidae	Well-known
Philodryas varia	Dipsadidae	Well-known
Philodryas viridissima	Dipsadidae	Well-known
Phimophis guerini	Dipsadidae	Well-known

Neotropical snakes	Families	Status of knowledge of geographical distribution
Phimophis guianensis	Dipsadidae	Well-known
Phimophis vittatus	Dipsadidae	Well-known
Phrynonax poecilonotus	Colubridae	Well-known
Phrynonax sexcarinatus	Colubridae	Well-known
Phyllorhynchus browni	Colubridae	Well-known
Phyllorhynchus decurtatus	Colubridae	Poorly-known
Pituophis catenifer	Colubridae	Poorly-known
Pituophis deppei	Colubridae	Well-known
Pituophis lineaticollis	Colubridae	Well-known
Plesiodipsas perijanensis	Dipsadidae	Poorly-known
Pliocercus elapoides	Dipsadidae	Well-known
Pliocercus euryzonus	Dipsadidae	Poorly-known
Porthidium dunni	Viperidae	Well-known
Porthidium lansbergii	Viperidae	Poorly-known
Porthidium nasutum	Viperidae	Well-known
Porthidium ophryomegas	Viperidae	Well-known
Porthidium porrasi	Viperidae	Poorly-known
Porthidium yucatanicum	Viperidae	Well-known
Pseudalsophis elegans	Dipsadidae	Well-known
Pseudelaphe flavirufa	Colubridae	Poorly-known
Pseudoboa coronata	Dipsadidae	Well-known
Pseudoboa haasi	Dipsadidae	Well-known
Pseudoboa martinsi	Dipsadidae	Well-known
Pseudoboa neuwiedii	Dipsadidae	Well-known
Pseudoboa nigra	Dipsadidae	Well-known
Pseudoboa serrana	Dipsadidae	Well-known
Pseudoeryx plicatilis	Dipsadidae	Well-known

Neotropical snakes	Families	Status of knowledge of geographical distribution
Pseudoeryx relictualis	Dipsadidae	Well-known
Pseudoficimia frontalis	Colubridae	Well-known
Pseudoleptodeira latifasciata	Dipsadidae	Well-known
Pseudotomodon trigonatus	Dipsadidae	Well-known
Psomophis genimaculatus	Dipsadidae	Well-known
Psomophis joberti	Dipsadidae	Well-known
Psomophis obtusus	Dipsadidae	Well-known
Ptychophis flavovirgatus	Dipsadidae	Well-known
Rena dulcis	Leptotyphlopidae	Poorly-known
Rena humilis	Leptotyphlopidae	Poorly-known
Rena maxima	Leptotyphlopidae	Well-known
Rena unguirostris	Leptotyphlopidae	Poorly-known
Rhachidelus brazili	Dipsadidae	Well-known
Rhadinaea calligaster	Dipsadidae	Well-known
Rhadinaea decorata	Dipsadidae	Well-known
Rhadinaea fulvivittis	Dipsadidae	Well-known
Rhadinaea gaigeae	Dipsadidae	Poorly-known
Rhadinaea hesperia	Dipsadidae	Poorly-known
Rhadinaea laureata	Dipsadidae	Poorly-known
Rhadinaea omiltemana	Dipsadidae	Poorly-known
Rhadinaea posadasi	Dipsadidae	Well-known
Rhadinaea pulveriventris	Dipsadidae	Poorly-known
Rhadinaea quinquelineata	Dipsadidae	Poorly-known
Rhadinaea taeniata	Dipsadidae	Well-known
Rhadinella godmani	Dipsadidae	Well-known
Rhadinella hannsteini	Dipsadidae	Poorly-known
Rhadinella hempsteadae	Dipsadidae	Well-known

Neotropical snakes	Families	Status of knowledge of geographical distribution
Rhadinella kanalchutchan	Dipsadidae	Poorly-known
Rhadinella kinkelini	Dipsadidae	Well-known
Rhadinella lachrymans	Dipsadidae	Poorly-known
Rhadinella montecristi	Dipsadidae	Poorly-known
Rhadinella pilonaorum	Dipsadidae	Poorly-known
Rhadinella schistosa	Dipsadidae	Poorly-known
Rhadinella seperaster	Dipsadidae	Poorly-known
Rhinobothryum bovallii	Colubridae	Poorly-known
Rhinobothryum lentiginosum	Colubridae	Well-known
Rhinocheilus lecontei	Colubridae	Well-known
Rodriguesophis chui	Dipsadidae	Well-known
Rodriguesophis iglesiasi	Dipsadidae	Well-known
Rodriguesophis scriptorcibatus	Dipsadidae	Well-known
Salvadora bairdi	Colubridae	Well-known
Salvadora deserticola	Colubridae	Poorly-known
Salvadora grahamiae	Colubridae	Poorly-known
Salvadora hexalepis	Colubridae	Poorly-known
Salvadora intermedia	Colubridae	Well-known
Salvadora lemniscata	Colubridae	Poorly-known
Salvadora mexicana	Colubridae	Well-known
Saphenophis boursieri	Dipsadidae	Poorly-known
Scaphiodontophis annulatus	Colubridae	Well-known
Scaphiodontophis venustissimus	Colubridae	Poorly-known
Scolecophis atrocinctus	Colubridae	Poorly-known
Senticolis triaspis	Colubridae	Well-known
Siagonodon acutirostris	Leptotyphlopidae	Poorly-known
Siagonodon borrichianus	Leptotyphlopidae	Poorly-known

Neotropical snakes	Families	Status of knowledge of geographical distribution
Siagonodon cupinensis	Leptotyphlopidae	Poorly-known
Siagonodon septemstriatus	Leptotyphlopidae	Poorly-known
Sibon annulatus	Dipsadidae	Poorly-known
Sibon anthracops	Dipsadidae	Poorly-known
Sibon carri	Dipsadidae	Poorly-known
Sibon dimidiatus	Dipsadidae	Well-known
Sibon longifrenis	Dipsadidae	Poorly-known
Sibon nebulatus	Dipsadidae	Well-known
Sibon sanniolus	Dipsadidae	Well-known
Sibynomorphus lavillai	Dipsadidae	Well-known
Sibynomorphus mikanii	Dipsadidae	Well-known
Sibynomorphus neuwiedi	Dipsadidae	Well-known
Sibynomorphus oneilli	Dipsadidae	Well-known
Sibynomorphus petersi	Dipsadidae	Poorly-known
Sibynomorphus turgidus	Dipsadidae	Well-known
Sibynomorphus vagus	Dipsadidae	Poorly-known
Sibynomorphus ventrimaculatus	Dipsadidae	Well-known
Simophis rhinostoma	Colubridae	Well-known
Siphlophis cervinus	Dipsadidae	Well-known
Siphlophis compressus	Dipsadidae	Well-known
Siphlophis leucocephalus	Dipsadidae	Well-known
Siphlophis longicaudatus	Dipsadidae	Well-known
Siphlophis pulcher	Dipsadidae	Well-known
Siphlophis worontzowi	Dipsadidae	Well-known
Sonora aemula	Colubridae	Poorly-known
Sonora michoacanensis	Colubridae	Poorly-known
Sordellina punctata	Dipsadidae	Well-known

Neotropical snakes	Families	Status of knowledge of geographical distribution
Spilotes pullatus	Colubridae	Well-known
Spilotes sulphureus	Colubridae	Well-known
Stenorrhina degenhardtii	Colubridae	Well-known
Stenorrhina freminvillei	Colubridae	Well-known
Storeria dekayi	Natricidae	Well-known
Storeria hidalgoensis	Natricidae	Well-known
Storeria storerioides	Natricidae	Well-known
Symphimus leucostomus	Colubridae	Poorly-known
Symphimus mayae	Colubridae	Well-known
Sympholis lippiens	Colubridae	Poorly-known
Synophis calamitus	Dipsadidae	Poorly-known
Synophis lasallei	Dipsadidae	Poorly-known
Tachymenis chilensis	Dipsadidae	Well-known
Tachymenis peruviana	Dipsadidae	Well-known
Taeniophallus affinis	Dipsadidae	Well-known
Taeniophallus bilineatus	Dipsadidae	Well-known
Taeniophallus brevirostris	Dipsadidae	Well-known
Taeniophallus nicagus	Dipsadidae	Well-known
Taeniophallus occipitalis	Dipsadidae	Well-known
Taeniophallus persimilis	Dipsadidae	Well-known
Taeniophallus poecilopogon	Dipsadidae	Well-known
Taeniophallus quadriocellatus	Dipsadidae	Well-known
Tantalophis discolor	Dipsadidae	Poorly-known
Tantilla alticola	Colubridae	Poorly-known
Tantilla armillata	Colubridae	Well-known
Tantilla atriceps	Colubridae	Poorly-known
Tantilla bocourti	Colubridae	Well-known

**Neotropical snakes**	**Families**	**Status of knowledge of geographical distribution**
Tantilla boipiranga	Colubridae	Well-known
Tantilla brevicauda	Colubridae	Poorly-known
Tantilla calamarina	Colubridae	Poorly-known
Tantilla cuniculator	Colubridae	Well-known
Tantilla deppei	Colubridae	Poorly-known
Tantilla impensa	Colubridae	Poorly-known
Tantilla jani	Colubridae	Well-known
Tantilla lempira	Colubridae	Poorly-known
Tantilla marcovani	Colubridae	Poorly-known
Tantilla melanocephala	Colubridae	Well-known
Tantilla miyatai	Colubridae	Poorly-known
Tantilla moesta	Colubridae	Poorly-known
Tantilla oaxacae	Colubridae	Poorly-known
Tantilla reticulata	Colubridae	Poorly-known
Tantilla rubra	Colubridae	Poorly-known
Tantilla schistosa	Colubridae	Well-known
Tantilla supracincta	Colubridae	Poorly-known
Tantilla tayrae	Colubridae	Poorly-known
Tantilla vermiformis	Colubridae	Poorly-known
Tantilla vulcani	Colubridae	Well-known
Tantilla wilcoxi	Colubridae	Poorly-known
Tantilla yaquia	Colubridae	Poorly-known
Tantillita canula	Colubridae	Poorly-known
Tantillita lintoni	Colubridae	Poorly-known
Thamnodynastes almae	Dipsadidae	Well-known
Thamnodynastes chaquensis	Dipsadidae	Well-known
Thamnodynastes gambotensis	Dipsadidae	Poorly-known

Neotropical snakes	Families	Status of knowledge of geographical distribution
Thamnodynastes hypoconia	Dipsadidae	Well-known
Thamnodynastes lanei	Dipsadidae	Well-known
Thamnodynastes longicaudus	Dipsadidae	Well-known
Thamnodynastes nattereri	Dipsadidae	Well-known
Thamnodynastes pallidus	Dipsadidae	Well-known
Thamnodynastes ramonriveroi	Dipsadidae	Well-known
Thamnodynastes rutilus	Dipsadidae	Well-known
Thamnodynastes sertanejo	Dipsadidae	Well-known
Thamnodynastes strigatus	Dipsadidae	Well-known
Thamnophis bogerti	Natricidae	Poorly-known
Thamnophis chrysocephalus	Natricidae	Well-known
Thamnophis cyrtopsis	Natricidae	Well-known
Thamnophis elegans	Natricidae	Well-known
Thamnophis eques	Natricidae	Well-known
Thamnophis fulvus	Natricidae	Well-known
Thamnophis godmani	Natricidae	Well-known
Thamnophis lineri	Natricidae	Poorly-known
Thamnophis marcianus	Natricidae	Well-known
Thamnophis melanogaster	Natricidae	Well-known
Thamnophis mendax	Natricidae	Well-known
Thamnophis nigronuchalis	Natricidae	Well-known
Thamnophis proximus	Natricidae	Well-known
Thamnophis rufipunctatus	Natricidae	Well-known
Thamnophis sauritus	Natricidae	Well-known
Thamnophis scalaris	Natricidae	Well-known
Thamnophis scaliger	Natricidae	Poorly-known
Thamnophis sumichrasti	Natricidae	Poorly-known

**Neotropical snakes**	**Families**	**Status of knowledge of geographical distribution**
Thamnophis valida	Natricidae	Well-known
Tomodon dorsatus	Dipsadidae	Well-known
Tomodon ocellatus	Dipsadidae	Well-known
Trachyboa boulengeri	Tropidophiidae	Poorly-known
Tretanorhinus nigroluteus	Dipsadidae	Well-known
Tretanorhinus variabilis	Dipsadidae	Well-known
Trilepida anthracina	Leptotyphlopidae	Poorly-known
Trilepida brasiliensis	Leptotyphlopidae	Well-known
Trilepida dimidiata	Leptotyphlopidae	Well-known
Trilepida fuliginosa	Leptotyphlopidae	Well-known
Trilepida jani	Leptotyphlopidae	Well-known
Trilepida joshuai	Leptotyphlopidae	Poorly-known
Trilepida koppesi	Leptotyphlopidae	Well-known
Trilepida macrolepis	Leptotyphlopidae	Well-known
Trilepida salgueiroi	Leptotyphlopidae	Well-known
Trimetopon gracile	Dipsadidae	Well-known
Trimetopon pliolepis	Dipsadidae	Well-known
Trimetopon slevini	Dipsadidae	Poorly-known
Trimorphodon biscutatus	Colubridae	Well-known
Trimorphodon tau	Colubridae	Well-known
Tropidodipsas annulifera	Dipsadidae	Well-known
Tropidodipsas fasciata	Dipsadidae	Poorly-known
Tropidodipsas fischeri	Dipsadidae	Well-known
Tropidodipsas philippii	Dipsadidae	Well-known
Tropidodipsas sartorii	Dipsadidae	Well-known
Tropidodryas serra	Dipsadidae	Well-known
Tropidodryas striaticeps	Dipsadidae	Well-known

Neotropical snakes	Families	Status of knowledge of geographical distribution
Tropidophis canus	Tropidophiidae	Well-known
Tropidophis grapiuna	Tropidophiidae	Well-known
Tropidophis haetianus	Tropidophiidae	Well-known
Tropidophis hendersoni	Tropidophiidae	Well-known
Tropidophis maculatus	Tropidophiidae	Well-known
Tropidophis melanurus	Tropidophiidae	Well-known
Tropidophis pardalis	Tropidophiidae	Well-known
Tropidophis paucisquamis	Tropidophiidae	Well-known
Tropidophis pilsbryi	Tropidophiidae	Well-known
Tropidophis preciosus	Tropidophiidae	Well-known
Tropidophis semicinctus	Tropidophiidae	Well-known
Tropidophis taczanowskyi	Tropidophiidae	Well-known
Tropidophis wrighti	Tropidophiidae	Well-known
Typhlophis squamosus	Anomalepididae	Well-known
Typhlops biminiensis	Typhlopidae	Well-known
Typhlops capitulatus	Typhlopidae	Well-known
Typhlops eperopeus	Typhlopidae	Well-known
Typhlops gonavensis	Typhlopidae	Well-known
Typhlops granti	Typhlopidae	Well-known
Typhlops hectus	Typhlopidae	Well-known
Typhlops hypomethes	Typhlopidae	Well-known
Typhlops lumbricalis	Typhlopidae	Well-known
Typhlops microstomus	Typhlopidae	Poorly-known
Typhlops monastus	Typhlopidae	Well-known
Typhlops platycephalus	Typhlopidae	Well-known
Typhlops pusillus	Typhlopidae	Well-known
Typhlops reticulatus	Typhlopidae	Poorly-known

**Neotropical snakes**	**Families**	**Status of knowledge of geographical distribution**
Typhlops rostellatus	Typhlopidae	Well-known
Typhlops schwartzi	Typhlopidae	Well-known
Typhlops sulcatus	Typhlopidae	Well-known
Typhlops syntherus	Typhlopidae	Well-known
Typhlops tetrathyreus	Typhlopidae	Well-known
Typhlops titanops	Typhlopidae	Well-known
Ungaliophis panamensis	Boidae	Poorly-known
Uromacer catesbyi	Dipsadidae	Well-known
Uromacer frenatus	Dipsadidae	Well-known
Uromacer oxyrhynchus	Dipsadidae	Well-known
Uromacerina ricardinii	Dipsadidae	Well-known
Urotheca decipiens	Dipsadidae	Poorly-known
Urotheca dumerilii	Dipsadidae	Poorly-known
Urotheca fulviceps	Dipsadidae	Poorly-known
Urotheca guentheri	Dipsadidae	Well-known
Urotheca lateristriga	Dipsadidae	Poorly-known
Urotheca multilineata	Dipsadidae	Poorly-known
Urotheca myersi	Dipsadidae	Well-known
Urotheca pachyura	Dipsadidae	Poorly-known
Xenodon dorbignyi	Dipsadidae	Well-known
Xenodon guentheri	Dipsadidae	Well-known
Xenodon histricus	Dipsadidae	Well-known
Xenodon matogrossensis	Dipsadidae	Well-known
Xenodon merremii	Dipsadidae	Well-known
Xenodon nattereri	Dipsadidae	Well-known
Xenodon neuwiedii	Dipsadidae	Well-known
Xenodon pulcher	Dipsadidae	Well-known

Neotropical snakes	Families	Status of knowledge of geographical distribution
Xenodon rabdocephalus	Dipsadidae	Well-known
Xenodon semicinctus	Dipsadidae	Well-known
Xenodon severus	Dipsadidae	Well-known
Xenodon werneri	Dipsadidae	Well-known
Xenopholis scalaris	Dipsadidae	Well-known
Xenopholis undulatus	Dipsadidae	Well-known
Xenopholis werdingorum	Dipsadidae	Well-known
