# Supplementary material for: Patterns, biases and prospects in the distribution and diversity of Neotropical snakes
Source: Glob Ecol Biogeogr. 2017 Nov 23;27(1):14–21. doi: 10.1111/geb.12679 (PMC5765514; doi:10.1111/geb.12679)
Supplement: Supplementary file 5 — Supporting Appendix S5 [file GEB-27-14-s005.pdf]

Patterns, biases and prospects in the distribution and diversity of Neotropical snakes

Thaís B. Guedes, Ricardo J. Sawaya, Alexander Zizka, Shawn Laffan, Alexander Pyron, Renato S. Bérnils, Martin Jansen, Paulo Passos, Ana L. C. Prudente, Diego F. Cisneros-Heredia, Henrique B. Braz, Cristiano de C. Nogueira & Alexandre Antonelli

Appendix S5 Table containing species richness summary in ecoregion scale. Specific species column show the number of species recorded exclusively in each ecoregion.

Ecoregion Name	Species Richness	Specific Species	Area of the Ecoregion (km^2)
Alta Paraná Atlantic forests	169	1	482879
Alvarado mangroves	3	0	40894
Amapa mangroves	0	0	17859
Apure-Villavicencio dry forests	49	1	68245
Araucaria moist forests	110	2	215673
Araya and Paria xeric scrub	21	0	5260
Argentine Espinal	42	0	298735
Argentine Monte	23	0	353640
Arid Chaco	21	0	99394
Aruba-Curacao-Bonaire cactus scrub	1	0	462
Atacama desert	1	0	104903
Atlantic Coast restingas	74	0	7850
Atlantic dry forests	68	0	114660
Bahamian dry forests	2	0	4827
Bahamian mangroves	4	1	26658
Bahamian pine forests	1	0	6870
Bahia coastal forests	106	1	109305
Bahia interior forests	130	1	229241
Bahia mangroves	31	0	8174
Bajío dry forests	13	1	37384
Balsas dry forests	28	1	62247
Belizean Coast mangroves	3	0	13461
Belizean Reef mangroves	0	0	10025
Belizian pine forests	15	0	2822
Beni savanna	35	0	125589
Bocas del Toro-San Bastimentos Island-San Blas mangroves	0	0	7827
Bolivian montane dry forests	43	3	72780
Bolivian Yungas	52	1	90229
Caatinga	102	5	731320
Caatinga Enclaves moist forests	41	0	4776
Campos Rupestres montane savanna	75	0	26313
Caqueta moist forests	35	1	183358
Catatumbo moist forests	6	0	22753
Cauca Valley dry forests	10	0	7313
Cauca Valley montane forests	19	2	31915

**Ecoregion Name**	**Species Richness**	**Specific Species**	**Area of the Ecoregion (km^2)**
Cayman Islands dry forests	0	0	134
Cayos Miskitos-San Andrés & Providencia moist forests	1	0	95
Central American Atlantic moist forests	44	5	89450
Central American dry forests	67	2	67773
Central American montane forests	47	4	13251
Central American pine-oak forests	84	7	110942
Central Andean dry puna	3	0	254929
Central Andean puna	6	0	211478
Central Andean wet puna	12	0	116948
Central Mexican wetlands	1	0	281
Cerrado	222	10	1910038
Chaco	83	1	786790
Chiapas Depression dry forests	23	0	13974
Chiapas montane forests	23	1	5759
Chilean matorral	2	0	148381
Chimalapas montane forests	10	0	2077
Chiquitano dry forests	123	3	229767
Chocó-Darién moist forests	35	2	73305
clipperton Island Scrub and Grassland	0	0	29
Coastal Venezuelan mangroves	11	1	5895
Cocos Island moist forests	0	0	25
Cordillera Central paramo	0	0	12120
Cordillera de Merida paramo	2	0	2798
Cordillera La Costa montane forests	33	0	14282
Cordillera Oriental montane forests	36	1	67577
Córdoba montane savanna	32	0	58472
Costa Rican seasonal moist forests	70	0	10900
Cuban cactus scrub	5	0	3256
Cuban dry forests	12	5	65745
Cuban moist forests	8	2	21339
Cuban pine forests	6	0	6405
Cuban wetlands	1	0	5647
Eastern Cordillera real montane forests	92	5	102062
Eastern Panamanian montane forests	2	0	3031
Ecuadorian dry forests	10	1	21187
Enriquillo wetlands	8	0	628
Esmeraldes/Chocó mangroves	4	0	6539
Everglades	0	0	20028
Fernando de Noronha-Atol das Rocas moist forests	0	0	19
Galapagos Islands xeric scrub	0	0	7978
Greater Antilles mangroves	18	1	10692
Guajira-Barranquilla xeric scrub	26	0	31477
Guayanan Highlands moist forests	77	3	145963
Guayaquil flooded grasslands	0	0	2924
Guianan Freshwater swamp forests	51	0	7690

Ecoregion Name	Species Richness	Specific Species	Area of the Ecoregion (km^2)
Guianan mangroves	72	0	14693
Guianan moist forests	108	0	476136
Gulf of Fonseca mangroves	1	0	1637
Gulf of Guayaquil-Tumbes mangroves	1	0	3343
Gulf of Panama mangroves	3	0	2445
Gurupa varzeá	7	0	9881
Guyanan savanna	78	0	104510
Hispaniolan dry forests	23	2	15446
Hispaniolan moist forests	21	3	45814
Hispaniolan pine forests	10	0	11560
Humid Chaco	102	0	291590
Humid Pampas	45	1	398555
Ilha Grande mangroves	45	0	3228
Iquitos varzeá	87	0	114506
Islas Revillagigedo dry forests	0	0	213
Isthmian-Atlantic moist forests	88	9	58359
Isthmian-Pacific moist forests	61	6	29176
Jalisco dry forests	49	2	26050
Jamaican dry forests	0	0	2310
Jamaican moist forests	0	0	8270
Japurá-Solimoes-Negro moist forests	77	0	268444
Juan Fernandez Islands temperate forests	0	0	146
Juruá-Purus moist forests	55	0	241493
La Costa xeric shrublands	47	0	68181
Lara-Falcón dry forests	19	1	16870
Leeward Islands dry forests	1	0	907
Leeward Islands moist forests	1	0	986
Leeward Islands xeric scrub	2	2	3228
Lesser Antilles mangroves	0	0	657
Llanos	49	0	375787
Madeira-Tapajós moist forests	128	1	716682
Magdalena Valley dry forests	17	1	19549
Magdalena Valley montane forests	40	1	104598
Magdalena-Santa Marta mangroves	9	0	3221
Magdalena-Urabá moist forests	12	0	76440
Magellanic subpolar forests	0	0	164642
Malpelo Island xeric scrub	0	0	8
Manabi mangroves	0	0	1159
Maracaibo dry forests	6	0	30085
Marajó Varzeá forests	84	0	88305
Maranhao Babaçu forests	69	0	141635
Maranhao mangroves	57	0	11414
Marañón dry forests	5	0	11322
Marismas Nacionales-San Blas mangroves	5	0	2054
Mato Grosso seasonal forests	123	1	412314
Mayan Corridor mangroves	0	0	4123
Mexican South Pacific Coast mangroves	4	0	1119

Ecoregion Name	Species Richness	Specific Species	Area of the Ecoregion (km^2)
Miskito pine forests	3	0	18854
Moist Pacific Coast mangroves	13	1	1611
Monte Alegre varzeá	75	0	66506
Mosquita-Nicaraguan Caribbean Coast mangroves	3	0	4473
Motagua Valley thornscrub	1	0	2328
Napo moist forests	100	3	250591
Negro-Branco moist forests	48	0	200932
Northeastern Brazil restingas	15	0	10011
Northern Andean paramo	10	0	29810
Northern Dry Pacific Coast mangroves	1	0	1065
Northern Honduras mangroves	0	0	1065
Northwestern Andean montane forests	57	11	80806
Oaxacan montane forests	20	1	7577
Orinoco Delta swamp forests	24	0	28028
Orinoco wetlands	6	0	5988
Panamanian dry forests	5	0	5087
Pantanal	85	0	170501
Pantanos de Centla	8	0	17153
Para mangroves	59	0	4452
Paraguana xeric scrub	5	0	15909
Paraná flooded savanna	48	0	37099
Patagonian grasslands	0	0	63184
Patagonian steppe	4	0	576599
Patia Valley dry forests	3	0	2261
Pernambuco coastal forests	44	2	17502
Pernambuco interior forests	56	1	22597
Peruvian Yungas	53	7	185961
Petén-Veracruz moist forests	70	3	148595
Petenes mangroves	5	0	1992
Piura mangroves	1	0	118
Puerto Rican dry forests	3	0	1271
Puerto Rican moist forests	7	0	7504
Purus varzeá	72	0	176760
Purus-Madeira moist forests	69	0	173261
Rio Lagartos mangroves	5	0	3491
Rio Negro campinarana	27	0	95986
Rio Negro-Rio San Sun mangroves	3	0	481
Rio Piranhas mangroves	30	0	2133
Rio Sao Francisco mangroves	34	0	2641
San Felix-San Ambrosio Islands temperate forests	0	0	6
San Lucan xeric scrub	0	0	3867
Santa Marta montane forests	8	1	4766
Santa Marta paramo	0	0	1239
Sechura desert	13	4	184213
Semi-arid Pampas	24	1	328370

Ecoregion Name	Species Richness	Specific Species	Area of the Ecoregion (km^2)
Serra do Mar coastal forests	136	2	104610
Sierra de la Laguna dry forests	0	0	3975
Sierra de la Laguna pine-oak forests	0	0	1061
Sierra de los Tuxtlas	8	0	3890
Sierra Madre de Chiapas moist forest	51	2	11218
Sierra Madre de Oaxaca pine-oak forests	22	3	14298
Sierra Madre del Sur pine-oak forests	28	6	60973
Sinaloan dry forests	48	12	77362
Sinú Valley dry forests	7	0	24879
Solimoes-Japurá moist forest	62	3	166931
South Florida rocklands	0	0	2071
Southern Andean steppe	9	0	124779
Southern Andean Yungas	32	0	75150
Southern Cone Mesopotamian savanna	58	0	26867
Southern Dry Pacific Coast mangroves	7	0	907
Southern Pacific dry forests	55	1	42282
Southwest Amazon moist forests	113	1	746653
St. Peter and St. Paul Rocks	0	0	6
Talamancan montane forests	69	5	16274
Tapajós-Xingu moist forests	98	0	335099
Tehuacan Valley matorral	12	0	9862
Tehuantepec-El Manchon mangroves	2	0	2716
Tepuis	21	1	50675
Tocantins/Pindare moist forests	101	1	192766
Trans-Mexican Volcanic Belt pine-oak forests	52	9	98111
Trindade-Martin Vaz Islands tropical forests	0	0	9
Trinidad and Tobago dry forests	11	0	5056
Trinidad and Tobago moist forests	40	1	4722
Trinidad mangroves	10	0	188
Tumbes-Piura dry forests	7	3	41100
Uatuma-Trombetas moist forests	109	0	470048
Ucayali moist forests	63	0	114443
Uruguayan savanna	67	0	352496
Usumacinta mangroves	0	0	3153
Valdivian temperate forests	3	0	248398
Venezuelan Andes montane forests	25	2	29269
Veracruz dry forests	13	0	6616
Veracruz moist forests	42	6	68946
Veracruz montane forests	4	0	4942
Western Ecuador moist forests	49	5	33954
Windward Islands dry forests	1	0	498
Windward Islands moist forests	4	2	2014
Windward Islands xeric scrub	0	0	1035
Xingu-Tocantins-Araguaia moist forests	91	0	265072
Yucatán dry forests	40	2	49623
Yucatán moist forests	42	2	69482
Zacatonal	2	0	303
